# Cell wall-driven mechanisms underlying emergent growth in phycomyces

**DOI:** 10.1007/s10237-026-02085-3

**Published:** 2026-07-10

**Authors:** Behnam Rezaei, Joseph K. E. Ortega, Franck J. Vernerey

**Affiliations:** 1 Department of Mechanical Engineering, The University of Colorado Boulder, Boulder, USA; 2 Department of Mechanical Engineering, University of Colorado Denver, Denver, CO 80217-3364 USA

**Keywords:** *Phycomyces blakesleeanus*, Cell wall, Helical growth, Morphogenesis, Mechanical model, Bond dynamics

## Abstract

Expansive growth and morphogenesis of plant and fungal cells are controlled by the mechanical properties of their cell wall. Despite extensive research, how the cell wall translates biochemical and environmental cues into anisotropic growth remains poorly understood. The sporangiophore (aerial hypha) of the filamentous fungus, *Phycomyces blakesleeanus*, exhibits remarkable behaviors, including rapid helical tip growth, light-stimulated growth responses, and handedness inversion during development. We present a mechanical model, informed by cell wall structure and experimental data, to account for these phenomena. The model incorporates anisotropic elasticity, viscoelastic creep from reversible bond dynamics, and tip extension via material deposition. Using this framework, we predict sporangiophore behavior in passive mechanical tests, including uniaxial stress relaxation and loading–unloading, and attribute features such as rotational inversion and light-stimulated growth responses to the interplay between fibril–tether network stiffness and bond kinetics. We further predict how changes in helical growth elements under altered turgor pressure correlate with the cell wall dynamics. Overall, our results show that fungal morphogenesis emerges from the mechanical properties of the cell wall rooted in its molecular organization.

**Supplementary Information:**

The online version contains supplementary material available at https://doi.org/10.1007/s10237-026-02085-3.

## Introduction

Plants and fungi continuously adapt their growth and form in response to environmental cues, a phenomenon that underlies their survival and developmental plasticity (Thellier 2017; Bahn et al. 2007). At the heart of this process is the ability of individual cells to convert physical and chemical signals into mechanical responses that drive morphogenesis. In walled cells, such as those of fungi and plants, this mechanical response is dominated by expansive growth, which results from the interplay between internal turgor pressure and the mechanical properties of the cell wall (Cosgrove 2000). Although the turgor pressure acts isotropically within the cell, it is the cell wall that determines how and where a cell deforms. The cell wall is far from being a passive envelope, instead it is a dynamic, anisotropic network of biopolymers whose structure and remodeling govern the mechanical behavior of the cell. The cell controls the mechanical properties of the wall in response to biochemical and environmental signals to direct anisotropic growth, but the precise relationship between wall architecture, mechanical response, and morphogenetic outcomes remains incompletely understood (Coen et al. 2023).

Deciphering the complexity of growth mechanisms in walled cells requires an integrative framework that captures the coupled dynamics of molecular kinetics, cell geometry, and turgor pressure driven mechanical deformation. At the molecular scale, the growth process is mediated by enzymatic and regulatory proteins that modify the architecture and composition of the cell wall, often through reaction-diffusion and kinetic processes (Chebli and Geitmann 2017; Archer and Peberdy 1997). These molecular events interact with geometric constraints and the elastic and permanent mechanical response of the wall to determine the spatial and temporal patterns of cell deformation. However, developing models that unify these disparate aspects is inherently challenging. Too often, mechanical models are tailored to reproduce a narrow set of observations, leading to inconsistencies when applied across different experimental contexts or scales (Cosgrove 2022). Moreover, while physical consistency and biological realism are critical, the models must remain sufficiently simplified to be interpretable and to yield predictive insights. The key, therefore, lies in combining the essential ingredients of growth, i.e., chemical, geometric, and mechanical, into a consistent theoretical framework that remains grounded in physical principles while accommodating biological complexity.

This work addresses this fundamental challenge by introducing a simple yet physically grounded model to explain the complex growth dynamics of the sporangiophore of *Phycomyces blakesleeanus* during development (Cohen and Delbrück 1959; Yoshida et al. 1980). The model is minimal in that it relies on only the essential parameters required to capture the observed behaviors, including viscoelasticity, helical growth, and anisotropy. The sporangiophore exhibits striking behaviors, including rapid helical tip growth (Ortega 1976; Ortega et al. 1974, 1988a), light-stimulated growth response (Ortega 1976), and handedness inversions during development (Russo and Galland 2005; Ortega et al. 2021; Goriely and Tabor 2011) that reflect intricate coupling between biochemical signaling, mechanical deformation, and cell wall remodeling. Despite decades of experimental investigation, the underlying mechanisms driving these behaviors remain elusive, in part due to the difficulty of disentangling the contributions of wall mechanics, cellular geometry, and dynamic protein activity.

Building on experimental observations and knowledge of the wall structure, we develop a minimal, physics-based model of the fungal cell wall that captures essential features of its chitin-rich network. Our model incorporates anisotropic elasticity, viscoelastic creep arising from reversible bond dynamics, and tip growth through material deposition at the tip. This framework allows us to quantitatively account for diverse growth behaviors and provides a mechanistic explanation for phenomena such as growth-induced twisting and developmental inversions. Equipped with this model, we compile and reinterpret a series of classical experimental observations spanning passive mechanical response, growth kinematics, and environmental adaptations. Using measured experimental data on helical growth elements, we determine the physiological ranges of the introduced parameters in our model, and show that the estimated values agree well with passive experimental results, including stress-relaxation and uniaxial loading-unloading experiments. After exploring the model predictions over a broad parameter space, we explain the mechanism governing the rotational inversion, light-stimulated growth response, and changes in helical growth elements under altered turgor pressure. Together, our results suggest that the morphogenesis of fungal cells can be understood as the emergent behavior of a dynamic, self-remodeling polymer network driven by turgor pressure. This work presents a unifying perspective on expansive growth through the integration of wall structure, mechanical behavior, and growth dynamics. Furthermore, this study highlights the central role of wall architecture in shaping cellular responses to the environment.

## Model description

### Experimental motivation

**Fig. 1:**
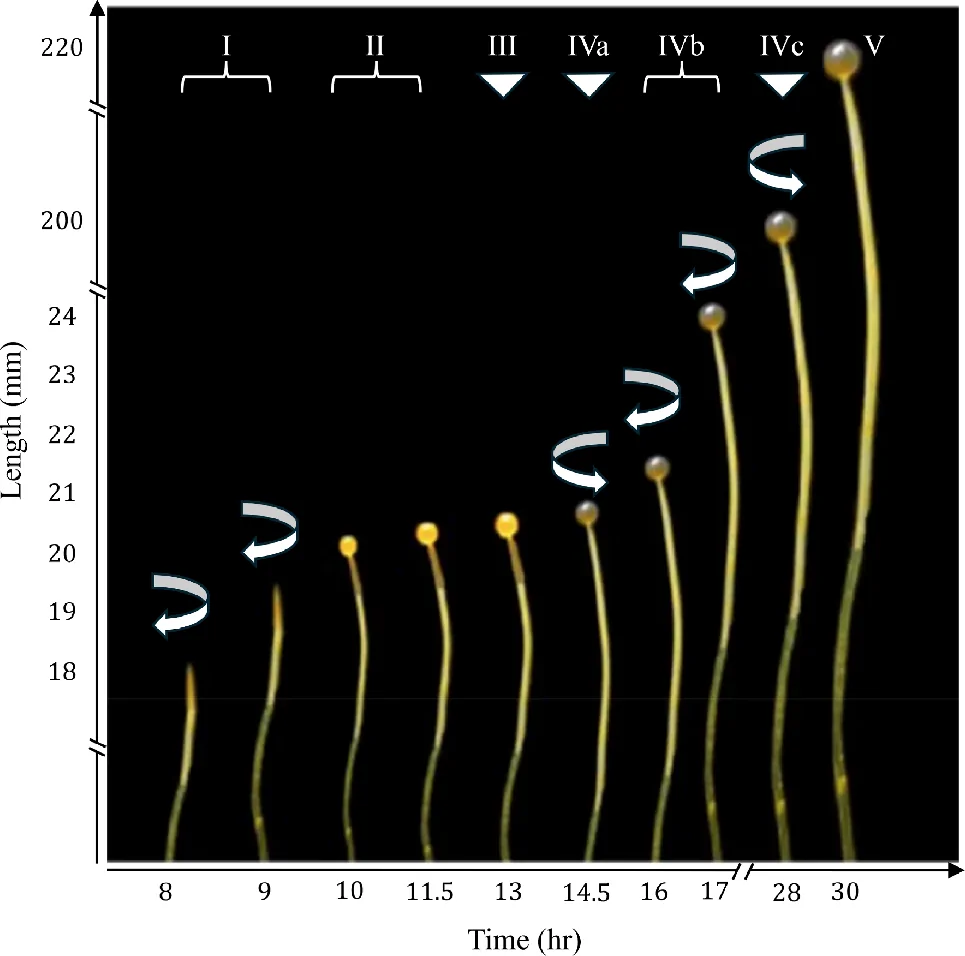
An illustration of five stages in the vegetative growth cycle of *Phycomyces blakesleeanus*. Left-handed helical growth (Stages I and IVb) and right-handed helical growth (Stages IVa and IVc) are indicated by clockwise and counterclockwise arrows (if viewed from above), respectively. The horizontal axis shows the time elapsed since the onset of growth, while the vertical axis corresponds to the sporangiophore length. [Figures were taken from Ortega et al. (2021) and reproduced with permission]

The sporangiophore (aerial stalk) of *Phycomyces blakesleeanus* is a single cell extending vertically from the mycelium, featuring a cell wall approximately $0.6 \; \mu m$ thick composed of an interconnected fibrous network. Embedded within this network are chitin microfibrils, approximately 150 - 250 $A^{\circ }$ in diameter, bound together by an amorphous matrix of chitin and chitosan tethers (Ortega 1976). Fig. 1 illustrates the five distinct stages of asexual growth in *P. blakesleeanus*. Expansive growth occurs in the "growth zone," located near the sporangiophore tip and extending downward from the sporangium (the sac-like structure at the apex). Experimental observations show that fibrils in the nongrowing stalk and lower growth zone adopt a steep spiral arrangement around the axial direction, with angles ranging from $1.5^{\circ }$ to $19^{\circ }$ in stage IVb. Conversely, fibrils in the upper growth zone exhibit less order and predominantly align transversely, with angles of $4^{\circ }$ to $15^{\circ }$ relative to the transverse direction (Roelofsen 1950a, b; Castle 1942; Heyn 1933; Roelofsen 1966; Mavis and Preston 1952). Stages I, II, and III exhibit left-handed helical growth, sporangium development, and spore formation, respectively. Due to its extended duration and rotational inversion phenomenon, stage IV is the most extensively studied in the literature. Stage IVa begins with right-handed helical growth (counterclockwise rotation viewed from above), lasting roughly one hour within a short growth zone located approximately 0.6mm below the sporangium. Toward the end of this stage, the elongation and rotation rates decrease, gradually transitioning into a clockwise rotation, which initiates stage IVb. Stage IVb, characterized by left-handed helical growth, lasts for approximately 30 – 48 hours. After a rapid increase in the elongation and rotation rates, the length of the growth zone, elongation rate, and rotation rate stabilize, at approximately $2.5 \; \text {mm}$, $35 \; \mu \text {m}. \text {min}^{-1}$, and $12^\circ \; \text {min}^{-1}$, respectively. Stage IVc commences with counterclockwise rotation, returning to right-handed helical growth. Stage V lacks visible growth (Ortega et al. 2021). The instantaneous inversion of rotation (from right-handed to left-handed and vice versa) during stage IV helical growth has been investigated by various researchers, leading to the proposal of several explanatory theories.

Wold (1992) fiber proposed a fiber-composite model linking the symmetric secretion of new fibers with the left-handed helical growth observed during stage IVb. By modeling a discontinuous array of fibers embedded in a matrix with power-law creep behavior, they accounted for variations in the rotation-to-elongation ratio along the sporangiophore. However, their model did not address the phenomenon of rotational inversion observed during stage IV. The “fibril reorientation” mechanism (Ortega and Gamow 1974; Ortega et al. 1974), later expanded into the “fibril reorientation-slippage” hypothesis (Ortega et al. 2003, 2021), attributes left-handed helical growth to the reorientation of fibers toward the axial direction under turgor pressure, and right-handed growth to inter-fiber slippage. Nonetheless, the slippage component remains qualitatively described and is primarily explained through geometric considerations. Goriely and Tabor (2011) introduced a continuum anisotropic model incorporating fiber deposition that successfully reproduces rotational inversion. Their model attributes the inversion to a transition from compressive prestress to tensile stress within the fibers. However, the prediction of individual material points rotating in both clockwise and counterclockwise directions within the growth zone is inconsistent with experimental findings (Gamow et al. 1986). Additionally, the two fitting timescale parameters central to their model lack correspondence with identifiable molecular mechanisms.

The cell wall thickness remains nearly constant during growth, indicating a continuous secretion of new materials from the protoplast (inside the cell). Additionally, new fibrils are added from the region below the sporangium, and the tip moves away from the deposited fibrils due to continued growth (Ortega and Welch 2013). The results from mechanical tests on the sporangiophore of stage IVb *P.blakesleeanus* suggest that the extensibility and permanent deformation are larger in the upper regions of the growth zone, those nearest the sporangium or apical tip (Norman et al. 1973). Thus, the upper regions of the growth zone demonstrate fluid-like behavior. Moving downward, the behavior of the cell wall more closely resembles that of an elastic solid. The fluid-like behavior suggests the dynamic nature of the cross-linked network inside the cell wall. The bonds between the tethers and fibrils can break and reform due to thermal fluctuation and the activity of enzymes, giving rise to dynamic behavior, such as creep and stress relaxation of the cell wall (Sridhar and Vernerey 2020; Sridhar et al. 2018). The variation in the rigidity and permanent deformation along the length of the sporangiophore can be attributed to the variation in both the stiffness and the crosslinking density of tethers (Vernerey et al. 2017; Vernerey 2022; Vernerey et al. 2024).

### Dynamic network model of the cell wall

**Fig. 2:**
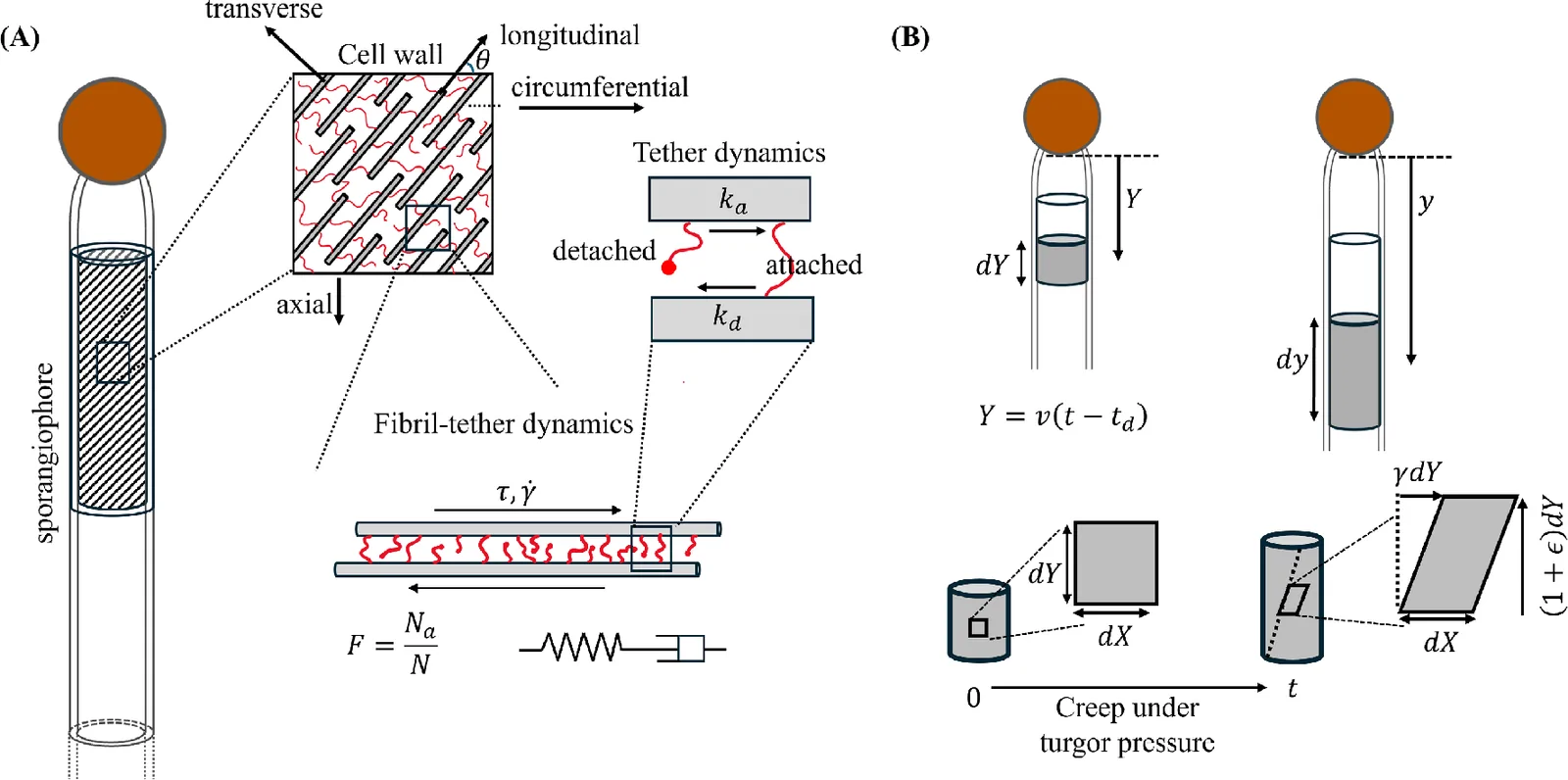
(A) Multiscale decomposition of the sporangiophore structure, indicating the cylindrical geometry (with constant radius), the anisotropic structure of the tether-fibril network, the role of the elastic tether population on the shear rheology of a system made of two fibrils subjected to a shear rate $\dot{\gamma }$, and with fraction *F* of connected fibrils, and finally, the dynamic behavior of an individual tether, exhibiting an association and dissociation rate $k_a$ and $k_d$, respectively. (B) Description of the history (here shown at an arbitrary time $t> t_d$) of a material element along the sporangiophore that is deposited at time $t_d$. The element is shown in the material frame (before deformation) and in the current frame (incorporating deformation). In its deformed state, this element exhibits a combination of axial strain ε and shear strain γ that fuel axial growth and rotation, respectively. We consider no deformation $\varepsilon _1 = 0$ in the hoop direction due to the constant radius

Fundamentally, our model is derived from the network description of the cell wall as a population of strongly aligned fibrils connected by dynamic tethers (Fig. 2A). Let us first concentrate on the population behavior of a total number $N = cL_f$ of tethers between two fibrils, where $L_f$ is the length of a fibril and *c* is the number density of available tethers (per unit length). The tethers are considered to be flexible chains with an end-to-end vector, whose projection onto the fibril direction is described by the random variable *r*. Consider these tethers as being connected to the fibrils by dynamic connections with association and dissociation rates $k_a$ and $k_d$, respectively. Using the standard transient network theory (Vernerey et al. 2017), it can be shown that the evolution of the mean end-to-end distance *r* of the full population when the fibrils undergo a global shear rate $\dot{\gamma }$ follows the coupled system first-order differential equations (See the supplementary materials):

2.1 $$\dot r = \dot{\gamma } - k_{a} \left( {\frac{{1 - F}}{F}} \right)r \qquad {\mathrm{and}} \qquad \dot{F} = k_{a} (1 - F) - k_{d} F{\text{ }}$$

where *F* is the fraction of the associated tethers in a population of $c_t$ tethers, with $c_t$ defined as the maximum number of available tethers per unit reference length. In other words, the linear density of the attached tether is given by $c_a = F c_t$. Further, using the simplifying assumption that the tethers are linearly elastic, with stiffness *K*, the stress over the entire population is given by $\tau = c_a K r = F c_t K r$. The above equation can thus be rewritten in terms of stress as:

2.2 $$\begin{aligned} \dot{\tau }+ k_d \tau \;=\; F c_t K \dot{\gamma }\end{aligned}$$

This is the governing equation of a Maxwell viscoplastic element with an equivalent shear modulus $G = c_t F K$ and shear viscosity $\eta = c_t F K/k_d$ (Fig. 2). This model can be generalized to a 2-dimensional element where the fibrils are oriented along a direction $\boldsymbol{a} = (\cos (\theta ), \sin (\theta ))$, which θ denotes the orientation angle of the fibrils relative to the horizontal axis, and for which the stiffness $E_f$ in the fibril direction is assumed to be independent of the tether concentration *c*. This gives:

2.3 $$\begin{gathered} \dot{\boldsymbol{\sigma}} + k_{d} \boldsymbol{\sigma} = \mathbb{C}(F) \cdot \dot{\boldsymbol{\varepsilon}} \qquad {\mathrm{and}} \qquad \dot{F} = k_{a} (1 - F) - k_{d} F \hfill \\ \end{gathered}$$

where the stress components ${\boldsymbol{\sigma }} = [\sigma _{1}, \sigma _{2}, \sigma _{12}]^T$ and the strain components ${\boldsymbol{\epsilon }} = [\varepsilon _{1}, \varepsilon _{2}, \epsilon _{12}]^T$ correspond to radial, axial and rotational growth deformation components, respectively. The matrix $\mathbb {C}$ is the classical elastic matrix for a transversely isotropic elastic network (Ting 1996a).

2.4 $$\begin{aligned} \mathbb {C}(F) = \begin{bmatrix} \left( E_f - F E_t\right) \cos ^4{(\theta )} + F E_t + E_v & \frac{1}{8} \left( E_f- F E_t \right) \left( 1 - \cos {(4 \theta )} \right) + E_v & \left( E_f - F E_t \right) \cos ^3 (\theta ) \sin (\theta )\\ \frac{1}{8} \left( E_f- F E_t \right) \left( 1 - \cos {(4 \theta )} \right) + E_v & \left( E_f - F E_t \right) \sin ^4{(\theta )} + F E_t + E_v & \left( E_f - F E_t \right) \cos (\theta ) \sin ^3 (\theta )\\ \left( E_f - F E_t \right) \cos ^3 (\theta ) \sin (\theta ) & \left( E_f - F E_t \right) \cos (\theta ) \sin ^3 (\theta ) & \left( E_f - F E_t \right) \sin ^2 (\theta ) \cos ^2 (\theta ) + \frac{F E_t}{2} \end{bmatrix} \end{aligned}$$

where $E_t$ is the maximum stiffness of tethers (assumed to be normal to the direction of fibers), which equals the stiffness *K* of one tether multiplied by the maximum available number $c_t$ of tethers $E_t = Kc_t$, and $E_v$ is the bulk modulus, which is a measure of the resistance to compression. Eq. (2.3) can be solved numerically to follow the evolution of stress and strain under various conditions.

### Spatiotemporal modeling of the growth mechanisms

The overall extensional growth of the sporangiophore arises from two processes: First, the deposition of new material at the base of the sporangium with a tip velocity *v*, at which fibrils are deposited at an angle $\theta _0$ slightly above the horizontal axis, and with very little connected tethers. Second, the constant creep of the wall material under the action of the turgor pressure $\textit{P}$. During this process, the radius of the sporangiophore and the wall thickness remain constant, implying the deposition of new material in the wall as it deforms. Given the constant radius, the strain components are reduced to $\boldsymbol{\epsilon }= [0, \varepsilon , \gamma ]^T$, which are shown in Fig. 2. Here, we aim to describe the axial strain ε and the shear strain γ as a function of the position from the sporangium. To define the concept of position, let us first introduce a so-called material coordinate *Y* of a point that was deposited at a time $t_d$ at the base of the sporangium. Note that this coordinate does not account for creep, but only for the continuous deposition of small layers of new wall material with infinitesimal width dY=vdt at the base of the sporangium Y=0. The material coordinate of a point deposited at time $t_d$ is therefore $Y(t;t_d) = \int _{t_d}^t v dt = v(t-t_d)$ , assuming that tip growth occurs at a constant rate *v*. This shows that the material coordinate of a material point translates away from the sporangium at velocity *v*. The true coordinates of this point must now incorporate the effect of creep. The deformed length *dy* of the material element deposited at time $t_d$ is given by $dy = (1 + \varepsilon (t_d)) dY$ where the strain $\varepsilon (t; t_d)$ arises from the axial creep of an element under the effect of turgor pressure $\textit{P}$ between times $t_d$ and *t*. The coordinates $x(t;t_d)$ and $y(t;t_d)$, which indicate the total shear deformation in the axial-circumferential plane and the distance from the sporangium, respectively, then result from the accumulation of these strains on elements deposited at a later time (i.e., $t>t_d$), i.e.:

2.5 $$\begin{gathered} x(t;t_{d} ) = v\int_{{t_{d} }}^{t} \gamma (t_{d} )dt \qquad \qquad y(t;t_{d} ) = v\int_{{t_{d} }}^{t} {\left( {1 + \varepsilon (t_{d} )} \right)} dt \hfill \\ \end{gathered}$$

where *x* describes the rotation, and *y* describes the elongation of points along the sporangiophore. The strains $\varepsilon (t;t_d)$ and $\gamma (t;t_d)$ of a material element that was deposited at time $t_d$, estimated at time $t> t_d$, can be determined by considering the evolution of the wall element under the action of turgor pressure $\textit{P}$. In this situation, the stress on the element remains constant ($\dot{\boldsymbol{\sigma }} = 0$, i.e., creep conditions) with $\sigma _{2} = PR_s/2t_w$, where $R_s$ and $t_w$ represent the radius of the sporangiophore and the thickness of the cell wall, respectively. Eq. (2.3) then turns into a simple evolution equation for the strain vector $\boldsymbol{\epsilon }$, which enables us to express the strain ${\boldsymbol{\epsilon }}(t;t_d)$ as the time integral:

2.6 $$\begin{gathered} {\boldsymbol{\epsilon}}(t;t_d) = \int_{t_d}^t \dot{\boldsymbol \epsilon} (t) dt \qquad \mbox{where} \qquad \dot{\boldsymbol{\epsilon}}(t) = k_d \mathbb{C}^{-1}(t) \cdot {\boldsymbol{\sigma}} \end{gathered}$$

subjected to the initial conditions $\theta (t_d) = \theta _0$ and $F(t_d) = 0$ (no tethers are connected to fibrils yet). We model growth under boundary conditions consistent with the experimental setup in which no external shear associated with fiber rotation is applied at the top of the stalk (free-rotating sporangium). According to the force equilibrium equation $\partial _y \sigma _{12} =0$, this results in the condition $\sigma _{12} = 0$ along the sporangiophore. We, however, note that in anisotropic elasticity, vanishing applied shear traction does not preclude the development of shear strain $\varepsilon _{12}$ (which is associated with fiber rotation). Regarding normal stresses, the axial component $\sigma _2$ is here prescribed by turgor pressure as discussed above, while the radial (hoop) stress $\sigma _1$ is determined such that it enforces the experimentally observed constant-radius condition $\varepsilon _1 = 0$ (Ortega et al. 1974). Physically, the maintenance of a constant radius is attributed to the load-bearing capacity of the second family of fibers, which are circumferentially oriented within the wall (Goriely and Tabor 2011). These fibers are distinct from those of the first family and exhibit a different response under creep, behaving in an elastic-like manner. This formulation is consistent with standard results for anisotropic cylinders, in which applied tractions and resulting strains are not in one-to-one correspondence (Ting 1996b).

Our model accounts for this by predicting a nonzero value for the circumferential stress $\sigma _{1}$. The fibril angle θ changes during the growth as follows:

2.7 $$\begin{aligned} \theta = tan^{-1}\left( \frac{(1+\epsilon ) sin \theta _0}{cos \theta _0 + \gamma sin \theta _0} \right) \end{aligned}$$

With the above kinetic equations, it is possible to predict both the cell wall structure and the dynamics of the sporangiophore during growth under initial conditions. The structure is provided by the fibril orientation $\theta (t;t_d)$ through Eq. (2.7) and the tether concentration $c(t;t_d)$. The growth dynamics are provided by the strain fields $\varepsilon (t;t_d)$ and $\gamma (t;t_d)$. Growth modeling is inherently a nonlinear problem. Material nonlinearities, arising from the fibrils’ reorientation and stretch, are accounted for by using the "updated Lagrangian" method (Yang et al. 2007), where updating the orientation of fibrils θ along with the density of attached tethers $Fc_t$ at each time step leads to updating the stiffness matrix $\mathbb {C}$ according to the deformed configuration. Nonlinear changes in geometry are also accounted for since wall thinning due to creep is balanced out by wall growth due to deposition of new fibrils from inside the protoplast (cell cytoplasm). Because of growth, the wall remains stable, and the geometrical nonlinearity does not play a large role. The simulations model the "growth" of the sporangiophore from the base of the sporangium to the end of the growth zone, which is defined as the point (or time) where the fibril direction nearly coincides with the vertical orientation. At this point, the wall material can no longer creep, and the model predicts vanishing elongation and rotation rates. To further simplify the analysis, we define the following dimensional variables: two time scales $\tau _T = \frac{1}{k_a}$ and $\tau _D = \frac{R_s}{v}$ related to tether and deposition dynamics, and one length scale $R_s$ defined by the radius of the sporangiophore and a stress-like quantity represented by the stiffness $E_f$ of the wall in the fibril direction. Another, more relevant characteristic length scale $L = v \tau _T$ arises from the competition between the velocity *v* of deposition and the time scale of tether dynamics. It will be a good reference to measure the size of the growth zone. Eventually, a general understanding of the fundamental mechanisms driving growth can be obtained through a non-dimensional analysis of the key drivers behind tip growth and mechanics. For our system, we identify four non-dimensional numbers:

2.8 $$\begin{aligned} & \sigma ^\star = \frac{pR_s}{2t_wE_f}, \qquad E^\star = \frac{E_t}{E_f}, \qquad L^\star = \frac{v}{R_s k_a} = \frac{L}{R_s} = \frac{\tau _T}{\tau _D}, \qquad \text{ and } \qquad K^\star = \frac{k_a}{k_a + k_d} \end{aligned}$$

Here, $\sigma ^\star$ is a measure of elastic deformation along the sporangiophore, $E^\star$ indicates the ratio of tether to fibril stiffness, $L^\star$ measures the competition between deposition-induced and creep-induced elongation, while $K^\star$ is the tether’s equilibrium constant that corresponds to the equilibrium fraction of tethers. With this definition, the dissociation rate constant is given by $k_d = \frac{1}{\tau _T} (\frac{1}{K^*} - 1)$.

**Table 1:** Model parameters used in the simulations and their estimated physiological ranges

Parameter	Symbol	Unit	Explanation	Physiological range (Stage IVb)
Detachment rate	$k_d$	$\text {min}^{-1}$	The rate of detachment of tethers from fibrils	0.05-0.3
Attachment rate	$k_a$	$\text {min}^{-1}$	The rate of attachment of tethers to fibrils	0.05-0.3
Fibril stiffness	$E_f$	$\text {MPa}$	The stiffness along the fibril direction	200-500
Max. tether stiffness	$E_t$	$\text {MPa}$	Maximum stiffness of tethers normal to fibril direction	100-400
Tip deposition rate	*v*	$\mu m/\text {min}$	Average rate of new wall material deposition at the tip	0.5-2

### Numerical implementation and model calibration

The numerical implementation of our model involves simulating creep under a constant turgor pressure for an element whose fibrils are oriented at an inclination angle $\theta _0$ relative to the horizontal axis, and mapping the element’s configuration at each time step to the spatial configuration of the sporangiophore. Creep is modeled using an updated Lagrangian approach, in which the body’s reference configuration is updated at each time step based on the deformed configuration from the previous step. Under this framework, employing a linear elasticity formulation between successive time steps is justified, provided that the time increment is sufficiently small such that the deformation between steps can be assumed linear. Furthermore, the material nonlinearity is assured in this method, as the effective elasticity matrix is updated at each time step (Yang et al. 2007). The steps for the numerical implementation of the steady-state growth model are described in Algorithm 1. This algorithm employs the Forward Euler method to numerically solve the differential equations in our model. Based on a convergence analysis, we selected a time step of dt=0.01s for the primary fitting procedure. Although the analysis indicated that larger time steps (dt>0.1s) yield elongation and rotation rates within 0.1% of those obtained with smaller steps for the chosen parameters, we adopted a finer time step to ensure higher accuracy, particularly in parameter sweep studies.

**Algorithm 1:**
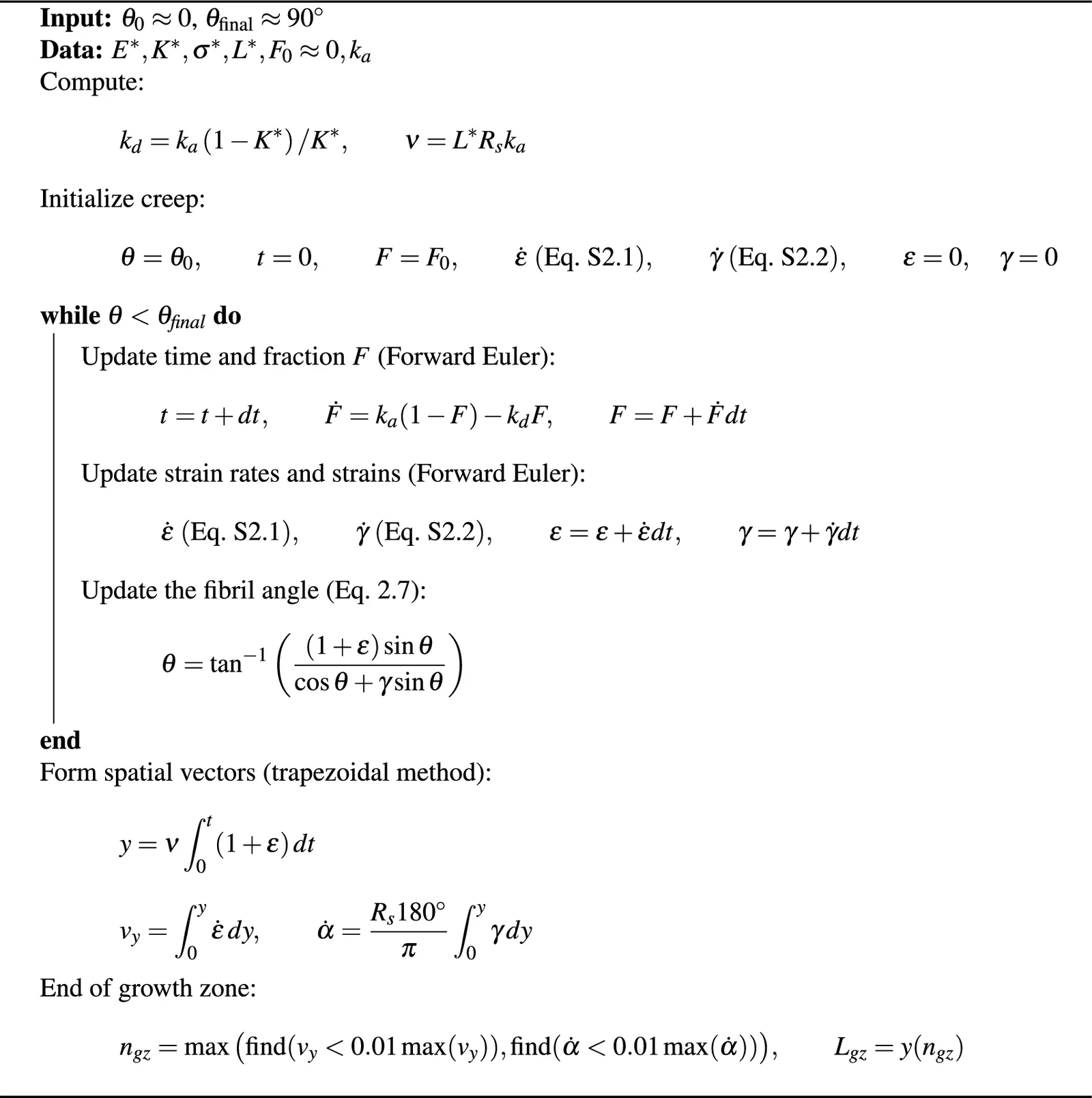
Numerical implementation of the growth model

Determining the parameters of the model is a nontrivial task, as the model serves two interconnected purposes: it generates the underlying structural organization of the sporangiophore, which must then be used to predict and compare mechanical responses with experimental data. To address this, we proceed sequentially. First, we use nondimensional parameters to construct a structure whose local growth behavior, characterized by elongation and rotation rates, matches experimental observations. At this stage, explicit values of forces are not required; only the relative contributions of the governing processes matter. Once a satisfactory structural configuration is achieved, we then evaluate its global mechanical response under controlled loading conditions (e.g., stress relaxation or cyclic tests). Comparison with experiments at this stage enables the identification of absolute material parameters, such as stiffness and bond kinetics, independently of the growth process.

**Fig. 3:**
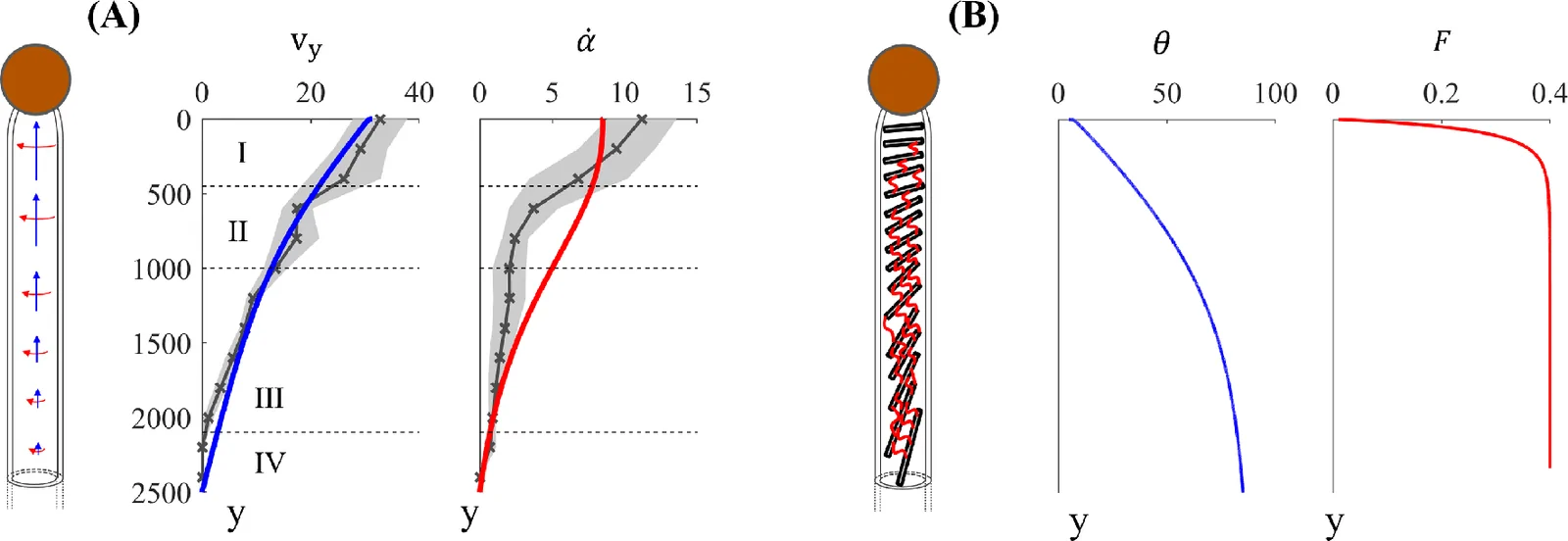
Calibration process for obtaining normalized values and comparing counterpart effects. (A) The elongation rate $v_y$ (μm/min) and the rotation rate $\dot{\alpha }$ ($(^{^{\circ }}/min)$) along the length of the growth zone as a function of the distance from the base of the sporangium y(μm). Shaded regions denote the standard deviation in experimental data (Ortega et al.1974a). Experiments shown in black, corresponding model results are shown in color. (B) Predicted fibril orientation θ with respect to the horizontal axis, and the fraction of attached tethers *F* within the tether population $c_t$ along the growth zone

The initial step involves determining the parameters that reproduce the experimentally observed structure from local measurements. Variations in elongation and rotation rates along the growth zone are characterized using the non-dimensional vertical stress $\sigma ^\star$ and non-dimensional tether stiffness $E^\star$, which capture their relative contributions to the overall growth behavior. In this context, Ortega (1976); Joseph et al. (1974a) used a conical mirror to measure the local elongation and rotation rates along the growth zone during stage IVb. Small markers, each with light-reflecting and light-absorbing surfaces on opposite sides, were placed on the sporangiophore. By positioning a light source at a 45-degree angle to the sporangiophore, they were able to measure rotation rates $\dot{\alpha }$ (in degrees per minute) and elongation rates $v_y$ (in μm per minute) at these locations. Fig. 3A displays the average measured values from these experiments (conducted on five different sporangiophores), along with their upper and lower bounds (mean ± standard deviation), plotted against the distance *y* from the sporangium. The data indicate an exponential-like decay in both elongation and rotation rates as the distance from the sporangium increases along the sporangiophore. However, the decay rate in the elongation and rotation rates in the upper region of the growth zone (approximately 450 μm below the sporangium) is minimal. In some of the experiments, the rates remained almost constant in this region (zone I). A sharp decrease in elongation and rotation rates is observed below the upper region, between about $450 \; \mu m$ and $1000 \; \mu m$ below the sporangium. However, the rate of decrease is more pronounced in the rotation rate values (zone II). The decay rate is slower between $1000 \; \mu m$ and $2000 \; \mu m$ from the sporangium (zone III). Finally, in the lower region of the growth zone, the rotation continues without significant elongation until the rotation itself also decreases to zero (zone IV).

We determine the admissible range of the non-temporal relative parameters by correlating the non-dimensional turgor stress $\sigma ^\star$ with the tip growth rate and the non-dimensional tether stiffness $E^\star$ with the ratio of rotation to elongation rates *R* along the sporangiophore. Moreover, the balance between material deposition and viscoelastic creep, used to predict the non-dimensional wall deposition rate $L^\star$, was constrained by the experimentally measured length of the growth zone. It is also important to note that, because the attachment and detachment kinetic rates of bonds are typically of the same order of magnitude, the equilibrium constant $K^\star$ (ranging between 0 and 1) does not approach either of these limiting values. As illustrated by the colored curves in Fig. 3A, the model reproduces the characteristic profiles of elongation ($v_y = \int _0^y \dot{\varepsilon }dy$) and rotation rates ($\dot{\alpha }= \int _0^y \dot{\gamma }dy$) observed experimentally along the sporangiophore. Although the model does not perfectly replicate all experimental values, it captures the overall trends, particularly in zone IV, where it successfully predicts a pronounced increase in the rotation to elongation ratio *R*. The value of non-dimensional parameters for our model prediction in Fig. 3A are $K^\star = 0.4$, $E^\star = 0.8$, $\sigma ^\star = 0.05$, and $L^\star = 0.172$. These values will serve as the basis for constructing the growth zone structure before modeling the viscoelastic response of the sporangiophore.

Given the lack of enough experimentally measured data points and considerable variations between conducted experiments, we do not aim to obtain an exact fit to the experimental data. Instead, we use manual parameter tuning for validation purposes. The focus is placed on reproducing qualitative growth trends, including changes in the ratio of rotation to elongation rates, the rates at which growth decreases, and the length of the growth zone. These trends, and their dependence on individual model parameters, are used to identify admissible parameter ranges. To assess the robustness of the fit, a sensitivity analysis was performed by perturbing the nondimensional turgor pressure $\sigma ^\star$, nondimensional tethers stiffness $E^\star$, and the equilibrium constant $K^\star$ by $\pm 10\%$ relative to their fitted values and evaluating the resulting changes in the model predictions (Fig. S2). While a 10% variation in the nondimensional parameters can lead to noticeable changes in the model predictions, the deposition rate *v* does not affect the creep response. Consequently, the nondimensional rate $L^\star$ may be tuned independently to match the predicted growth zone length $L_{gz}$ with the experimentally measured length. As a result, multiple qualitative fits to the same experimental dataset can be obtained using different parameter combinations. For example, Fig. S3 shows three qualitatively similar fits to the same growth rate data obtained with different values of the equilibrium constant $K^\star$ and nondimensional deposition rate $L^\star$.

The observed variation in rigidity and plastic deformation during passive tests, as well as the rotation-to-elongation rate ratio during steady-state active growth along the sporangiophore, can be attributed to wall anisotropy. In our model, this anisotropy, i.e., reflecting differences in mechanical properties across directions, arises from changes in fibril orientation and in the density of attached tethers along the sporangiophore. Thus, we study how our model predicts these variations. Fig. 3B shows the distribution of fibril orientation θ with respect to the horizontal axis, and the non-dimensional transverse stiffness $FE^\star$ along the length of the growth zone. The fibrils are predicted to reorient progressively, from nearly transverse below the sporangium ($\theta \approx 0$) to nearly vertical ($\theta \approx 90^\circ$) at the end of the growth zone. The predicted reorientation is consistent with experimental studies (Roelofsen 1950a, b; Castle 1942; Heyn 1933; Roelofsen 1966; Mavis and Preston 1952). The length of the growth zone $L_{gz}$ is identified as the material point where the elongation and rotation rates decay to zero (practically defined as a small fraction of their tip values). The model further predicts that the stiffness ratio between the transverse (normal to fibril orientation) and longitudinal (parallel to fibril orientation) directions, $FE^\star$, increases along the sporangiophore axis. This trend is governed by the kinetic rates $k_a$ and $k_d$ Eq. (2.1), while the final value of $FE^\star$ is set by the equilibrium constant and equals $K^\star E^\star$. The elongation and rotation rates at each point along the sporangiophore represent the cumulative contributions of the axial deformation rate $\dot{\varepsilon }$ and the shear deformation rate $\dot{\gamma }$ from all material points below. To further elucidate the model’s behavior, analyses at the level of individual points are presented in the following sections.

We assumed constant kinetic rates, i.e., the attachment rate $k_a$ and the detachment rate $k_d$, along the length of the growth zone during growth. However, stress-relaxation experiments on the sporangiophore (Section 2.5) indicate that these rates are not constant but instead depend on the forces borne by the bonds, which arise from turgor pressure. Although incorporating force-dependent kinetic rates is beyond the scope of the present study, additional analysis suggests that while such force dependence alters the values of other parameters required to fit the experimental data, the overall trends in the predicted growth rates remain similar to those obtained using constant rates.

### Sporangiophore viscoelasticity

To assess the passive mechanical response of the entire sporangiophore, experiments were performed under applied loads. Here, we use these data to identify the physiological ranges of the model parameters, as summarized in Table 1. We then use the structure obtained from the previous section to predict, compare, and finally calibrate the parameters that govern the time-dependent mechanical response of the sporangiophore.

**Fig. 4:**
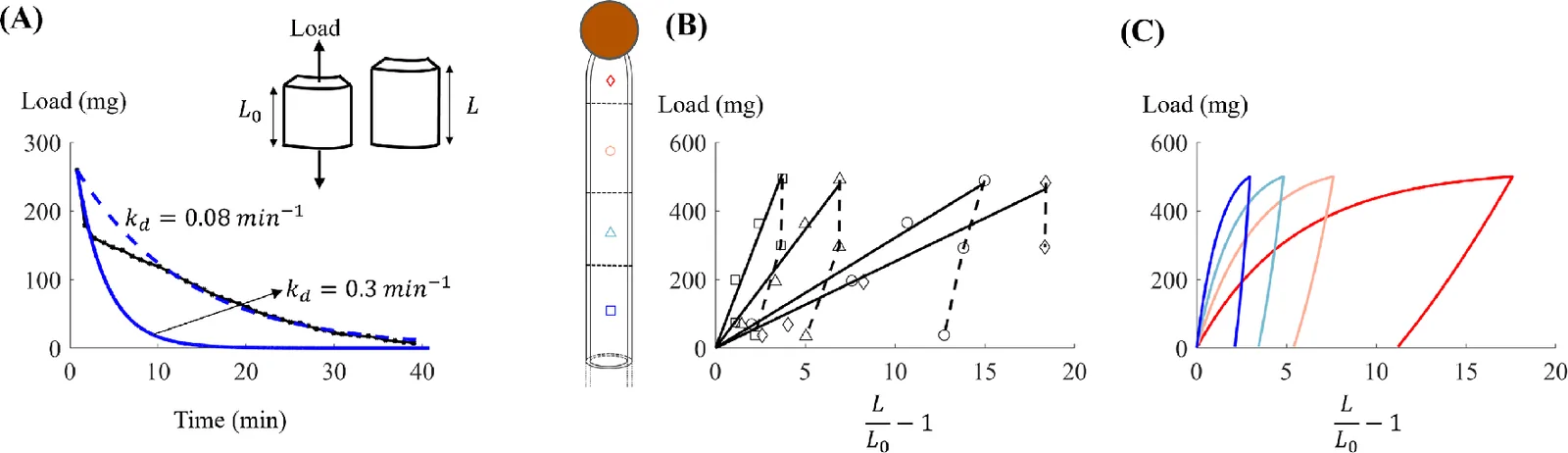
Experimental observations and model predictions on the stage IVb sporangiophore of *P. blakesleeanus*. All experiments shown in black; corresponding model results are shown in color. (A) Load (mg) vs. time (min) in a stress relaxation test of the stage IVb sporangiophore following uniaxial loading (Ortega 1976). Solid and dashed curves represent model predictions for two limiting values of dissociation rate $k_d$. (B, C) Load (mg) vs engineering strain ($L/L_0 - 1$) for four regions (denoted by markers) on the growth zone in a uniaxial loading-unloading experiment (Norman et al. 1973). These simulations serve as a means of determining the absolute values of the bond kinetic parameters

*Stress relaxation. * (Ortega^1976^) reported uniaxial tensile stress relaxation tests on stage IVb *P. blakesleeanus* sporangiophores. In these experiments, the tip was rapidly extended at a rate of 1 mm/min to a load of 260 mg, after which the deformation was held constant while the resulting force was recorded. In this test, the deformation is applied at a rate that is faster than growth and thus may be used to separate the evaluation of intrinsic material parameters (bond dynamics, elasticity parameters), independently from the active growth process. Fig. 4A compares the above experimental data (black) with the corresponding model prediction (blue). The measured force exhibits an approximately exponential decay, with a faster decay rate observed at the beginning of the experiment. In our model, the decay of force during stress relaxation depends solely on the bond dissociation rate, $k_d$, due to the assumption that bonds reattach in a stress-free configuration. To replicate the experimental stress relaxation behavior, we find that the detachment rate of $k_d = 0.3 \; \text {min}^{-1}$ predicts the fast decay of the first few minutes of the experiment. However, the decay rate of the rest of the relaxation experiment (after about 10 minutes) matches the detachment rate of $k_d = 0.08 \; \text {min}^{-1}$. This variation accounts for the dependence of bond kinetics on elastic stretch, a relationship that was described by various forms in the literature (Bell 1978; Bird et al. 1987; Lamont and Vernerey 2022; Lin et al. 2023; Samuel et al. 2025; White et al. 2025). Given that the external stress on the sporangiophore progressively vanishes by the end of the relaxation experiment, the physiological, steady-state value of $k_d$ is likely near the lower limit of the estimated range. The model successfully captures the overall exponential decay profile observed in the experiment. However, reproducing the initial rapid drop in force would require a force-dependent dissociation rate, i.e., $k_d = k_d(f)$. For simplicity, the current model uses a constant $k_d$ along the length of the sporangiophore and over time.

*Uniaxial loading-unloading.* Uniaxial loading experiments are conducted to assess the rigidity of cell walls and are followed by an unloading experiment to determine the permanent deformation (Norman et al. 1973). We use our model to compare its predictions with the experimental measurements of Norman et al. (1973) in a loading-unloading experiment (Fig. 4B). We utilize the fibril orientation θ and the ratio of attached crosslinkers *F* from the steady-state growth modeling, and we construct the initial configuration of the sporangiophore before loading. Assuming a uniform radius and wall thickness, the stress distribution is uniform along the sporangiophore. We determine the time derivative of the stress to ensure that the tip speed, which corresponds to the cumulative sum of the speeds of each element along the sporangiophore, matches the experimentally imposed tip speed of $254 \; \mu m/min$ throughout the test (the tip speed was the same in loading and unloading phases). Since the speed tip is much larger than the tip elongation rate, we can neglect the effect of deposition in these experiments. By defining four distinct regions within the growth zone, we compute the engineering strain (the relative length change of these regions) at each applied load.

Fig. 4C illustrates the predicted force-strain behavior of the four defined regions along the sporangiophore. These results were obtained using a higher bond dissociation rate $k_d$, than that employed in the steady-state growth modeling. The reason behind this adjustment is the dependence of the bond dissociation rate on elastic stretch, as mentioned before. Our model predicts that in the upper region of the sporangiophore, more than half of the deformation is elastic. Given the significant elastic stretch observed in this test, the kinetic rates were adjusted accordingly. For simplicity, we assume these rates remain uniform across both space and time. The model successfully captures key experimental trends: both extensibility and permanent deformation upon unloading decrease progressively from the upper to the lower regions of the growth zone. However, some discrepancies remain between our predictions and experimental data. The experimental stress–strain curves display linear behavior during loading. To reproduce this, the model must account for fibril strain-hardening as they are stretched, with the overall stress–strain response governed by the balance between strain-hardening and bond dynamics, the latter potentially leading to strain-softening. We, however, used a constant fibril stiffness to avoid more complexities. Furthermore, another likely source of this discrepancy is that the region lengths in our model do not precisely match those defined in the experiments due to the limited data available in Norman et al. (1973).

## Analysis of growth dynamics and stimulus response

The helical growth of the *P. blakesleeanus* sporangiophore is sensitive to changes in turgor pressure and to external stimuli such as gravity, mechanical obstruction (avoidance response), and light. Responding to these stimuli, the helical growth components, namely the elongation and rotation rates, as well as the length of the growth zone, deviate from their steady-state values. Before applying our model to predict the sporangiophore’s response to these stimuli, we first analyze its behavior over a broad range of parameters to gain deeper insight into the emergent dynamical growth characteristics and their underlying physical mechanisms. Subsequently, we summarize key experimental studies investigating the effects of altered turgor pressure (pressure response) and light stimulation on sporangiophore helical growth and use our model to interpret the observed responses in terms of their governing physical processes.

**Fig. 5:**
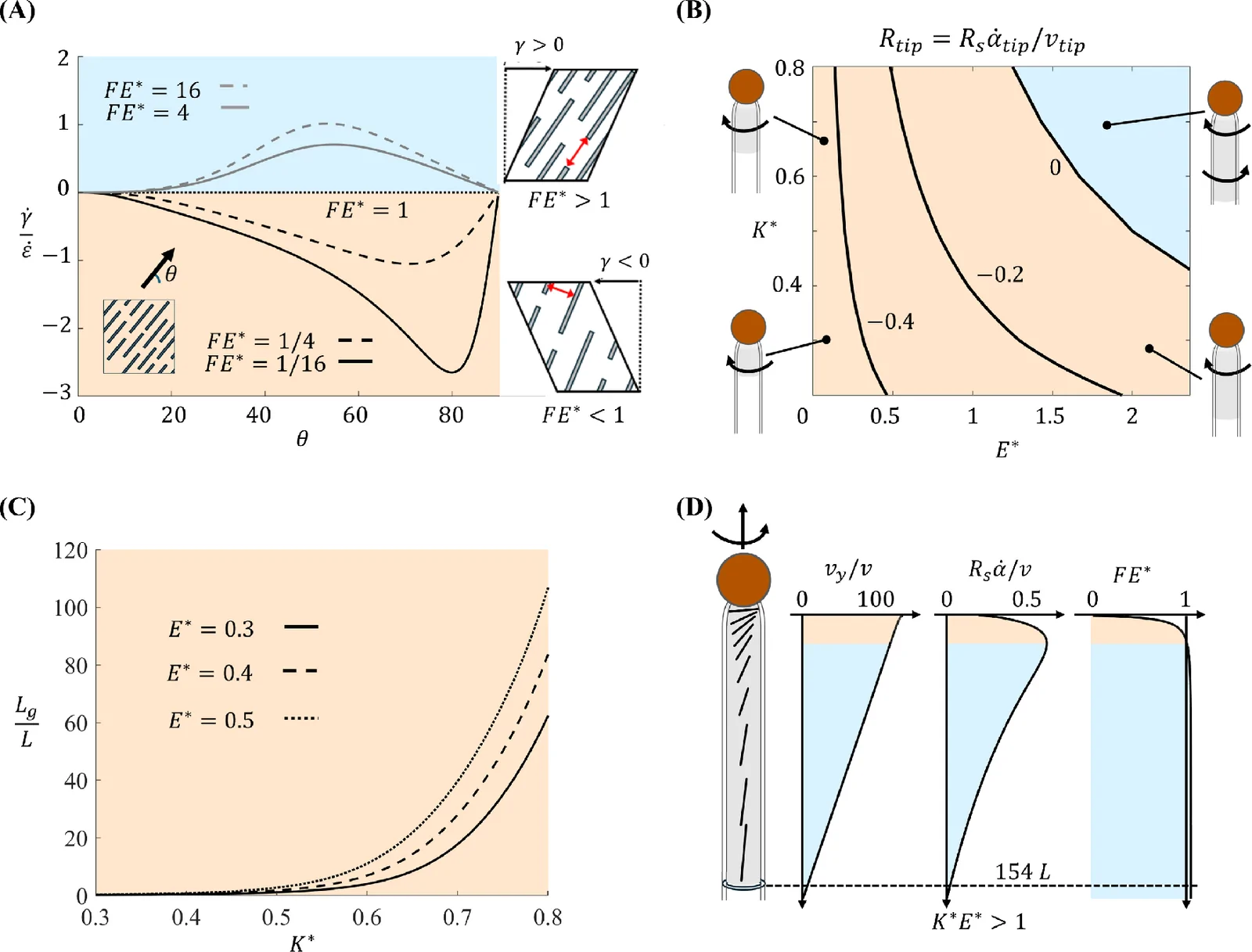
(A) The shear to axial strain rate ratio $\dot{\gamma }/\dot{\varepsilon }$ as a function of fibril orientation θ for $FE^\star = 1/16, \; 1/4, \; 1, \; 4 \; and \; 16$. The schematics show the "fibril lengthening dominated" (top) vs "fibril separation dominated" (bottom) mechanisms for positive shear (γ>0 and $FE^\star> 1$) and negative shear (γ<0 and $FE^\star < 1$), respectively. (B) A contour map of the tip normalized rotation-to-elongation ratio $R_{tip} = R_s \dot{\alpha }_{tip/v_{tip}}$ as a function of non-dimensional tethers stiffness $E^\star$ and non-dimensional equilibrium constant $K^\star$, showing a transition from counter-clockwise to clockwise rotation. Schematics show the associated growth zones (the relative length and the twist direction). (C) Non-dimensional length of the growth zone $L_{gz}/L$ as a function of non-dimensional equilibrium constant $K^\star$ for $E^\star = 0.3, \; 0.4, \; and \; 0.5$. (D) Model predictions of wall structure, growth zone length $L_g$, non-dimensional tip rotation rate $R_s \dot{\alpha }/v$, non-dimensional tip speed $v_y/v$, and the non-dimensional transverse stiffness $FE^\star$ along the sporangiophore for the right-handed helical growth. $K^\star = 0.52$, $E^\star = 2$, $\sigma ^\star = 0.9$, and $L^\star = 0.1$. (A). The orange and blue backgrounds correspond to left-handed and right-handed helical growth, respectively

### Rotational inversion

Previous studies have proposed various theories to explain the rotational inversion observed during stage IV (Ortega et al. 2021; Goriely and Tabor 2011; Ortega and Gamow 1974; Ortega et al. 2003). As mentioned earlier, the direction of rotation during helical growth (clockwise vs. counterclockwise) is directly linked to the direction of shear deformation γ in a wall element. A negative shear rate corresponds to left-handed helical growth, while a positive shear rate results in right-handed helical growth. By combining Eqs. (2.3) and (2.4), and applying the growth condition, i.e., constant radius and constant stress (See the supplementary materials), the analytical expression for the shear to axial deformation is calculated as:

3.1 $$\begin{aligned} \dot{\gamma }/ \dot{\varepsilon }= \frac{\left( F E^\star - 1 \right) \left( \sin (2\theta ) - \cos (2\theta ) \sin (2\theta ) \right) }{ \sin ^2(2\theta ) + F E^\star (1+ \cos ^2(2\theta ))} \end{aligned}$$

Thus, the non-dimensional transverse stiffness $FE^\star$ determines the magnitude and direction of rotation as well as the rotation rate to elongation rate ratio. Fig. 5A illustrates the changes in the shear to axial strain rate ratio, $\dot{\gamma }/\dot{\varepsilon }$ vs. the fibril orientation θ for five different values of non-dimensional transverse stiffness $FE^\star$. A non-dimensional stiffness $FE^\star = 1$ represents an isotropic network, which shows no rotation. If the stiffness along the fibrils is greater than the transverse stiffness ($FE^\star < 1$), the model predicts a negative shear deformation, corresponding to left-handed helical growth. Conversely, if the transverse stiffness exceeds the longitudinal stiffness ($FE^\star> 1$), a positive shear deformation occurs, leading to right-handed helical growth. Thus, the handedness of the rotation depends on the relative stiffness, regardless of whether the bond kinetics are force-dependent. The primary mechanism governing the direction of rotation can be linked to the deformation mode of the interconnected fibril network. In a left-handed growing element, the dominant mode is the separation between parallel fibrils, whereas in a right-handed growing element, the elongation of individual fibrils prevails. We refer to these modes as ‘fibril-separation–dominated’ and ‘fibril-lengthening–dominated,’ respectively, as illustrated in the schematics of Fig. 5A. It should be emphasized that both fibril elongation and separation occur in each mode; however, their relative dominance differs. For instance, consider a wall element with fibrils initially oriented at an angle $\theta _0$ relative to the horizontal axis. Let $l_0$ represent the initial average fiber length, and $\delta _0$ the initial average spacing between parallel fibers. Under deformation produced by turgor pressure, the wall element adopts a new orientation, resulting in changes to both fibril length and spacing. Although both positive and negative shear strains γ result in elongated and more distantly spaced fibers, the extent of these changes depends on the direction of shear. Assuming $l^+$ and $\delta ^+$ denote the average fiber length and spacing under positive shear strain, and $l^-$ and $\delta ^-$ represent these values under negative shear strain (of equal magnitude), our model predicts that right-handed helical growth corresponds to greater fiber elongation, $l^+> l^-> l_0$, while left-handed helical growth results in greater fiber spacing, $\delta ^-> \delta ^+> \delta _0$.

The observed inversion from right-handed growth in stage IVa to left-handed growth in stage IVb can thus be attributed to the evolution of the relative transverse stiffness $FE^\star$. In stage IVa, newly deposited fibrils lack sufficient maturity, and the transverse stiffness remains dominant, leading to right-handed growth. As the sporangiophore matures and fibrils develop, their stiffness in the longitudinal direction surpasses the transverse stiffness, resulting in a transition to left-handed growth in stage IVb. With continued material deposition and bond formation, the transverse stiffness may eventually exceed the longitudinal stiffness again at the end of stage IVb, leading to a second rotational inversion back to right-handed growth in stage IVc. For clarity of the exposition, the calculations of the maximum value of $\dot{\gamma }/\dot{\varepsilon }$ and its associated fibril orientation are not shown here but are available in the supplementary materials. As mentioned earlier, the tip growth speed $v_{tip}$ and the tip rotation rate $\dot{\alpha }$ are the cumulative values of the elongation and rotation rates of all the material points on the growth zone. Thus, the ratio of the tip rotation to elongation rate is a function of the variation of the non-dimensional transverse stiffness $FE^\star$. The phase diagram in Fig.5B illustrates the non-dimensional tip rotation-to-elongation rate, $R_{tip} = R_s \dot{\alpha }_{tip}/v_{tip}$, across various values of non-dimensional tether stiffness $E^\star$ and non-dimensional equilibrium constant $K^\star$. Negative values of $R_{tip}$ indicate left-handed helical growth (orange background), while positive values correspond to right-handed helical growth at the tip (light blue background). For all the cases shown, $F_0 E^\star < 1$, indicating that the upper part of the growth zone experiences greater deformation, i.e., fluid-like behavior. Note that a negative value of $R_{tip}$ does not necessarily mean that all material points rotate in the same direction. This nuance is illustrated in the four schematic sporangiophores shown in Fig. 5B. In the example corresponding to the blue region, material points near the top of the growth zone rotate clockwise, while those at the bottom rotate counterclockwise.

### Growth zone and its role in mediating rotation

Another measurable parameter frequently reported in the literature is the length of the growth zone $L_{gz}$. This length varies across different growth stages and in response to external stimuli such as light. For example, the growth zone length is approximately 0.6mm during stage IVa and increases during the transition to stage IVb until it approaches a steady-state length around 2.5 - 3 mm for most of the stage. Moreover, the growth zone extends further in response to light stimulation and other sensory stimuli. Experimentally, the boundary of the growth zone is identified by the point beyond which no measurable elongation or rotation is observed on the stalk. In contrast, our simulations define the end of the growth zone based on a criterion where elongation and rotation rates reach a final threshold value (e.g., one percent of their maximum). Given the presence of points in experimental data with measurable rotation but negligible elongation (steady-state growth) and vice versa (light-stimulated), we select the criterion resulting in the longest growth zone, as described in Algorithm 1. Alternative criteria for determining the length of the growth zone include: (i) selecting the minimum length at which only one of the fractional conditions is satisfied, and (ii) defining the growth zone length as the position of the last simulated material point corresponding to a fibril angle of $\theta _{\text {final}}$. In all cases, the predicted growth zone length remains close to that obtained using the primary criterion adopted in this study. A comparison of the predicted growth zone lengths under these different definitions is presented in Fig. S4 (supplementary materials). Fig. 5C depicts the variation of non-dimensional growth zone length $L_{gz}/L$ with the non-dimensional equilibrium constant $K^\star$ for three distinct values of non-dimensional tether stiffness $E^\star$. The length of the growth zone increases with higher values of both $K^\star$ and $E^\star$, indicating that the increased stiffness of the network, whether due to a higher $K^\star$ or $E^\star$, prolongs fiber reorientation, resulting in a longer growth zone. Furthermore, the length of the nondimensional growth zone $L_{gz}/L$ remains unaffected by variations in the nondimensional length $L^\star$, which shows the competition between deposition and creep rates. The same trends are observed when using any of the alternative criteria for determining the growth zone length.

The changes associated with an increased equilibrium constant $K^\star E^\star$, including the extended growth zone length and the inversion of rotation, are illustrated in Fig. 5D. The figure presents the distributions of fibril orientation θ (black fibrils shown on the sporangiophore), the non-dimensional elongation rate $v_y/v$, the non-dimensional rotation rate $R_s \dot{\alpha }/v$, and the non-dimensional transverse stiffness $FE^\star$ along the growth zone for a sporangiophore exhibiting greater transverse stiffness at the growth zone end ($K^\star E^\star> 1$). A transition in wall anisotropy occurs about 15*L* below the sporangium, where stiffness shifts from being greater along the fibril direction ($FE^\star < 1$) to greater in the transverse direction ($FE^\star> 1$). Above this point, material points rotate clockwise, whereas below it they rotate counterclockwise, yielding a net counterclockwise rotation at the tip. If, however, the transverse stiffness was greater along the entire sporangiophore ($FE^\star> 1$), our model yields higher axial strain ε at the base of the growth zone (below the sporangium) than at its tip. This implies an unrealistic distribution of material behavior, with solid-like properties at the tip and fluid-like behavior at the base, contradicting experimental observations. We therefore excluded this case from further analysis.

### Pressure response

Turgor pressure, the driving force of the walled cells growth, plays an important role in determining the growth rates. The "stress relaxation–water uptake" mechanism, originally proposed by Cosgrove (1993a, 1993b), explains how growth in plants and fungi is facilitated by the coupled processes of osmotic water uptake and stress relaxation of the cell wall through the breakage of load-bearing bonds. According to this mechanism, wall stress relaxation produces a reduction in intracellular pressure. This reduction, in turn, promotes additional water uptake, thereby restoring turgor pressure and sustaining continued growth. Ortega provided a mathematical formulation of this mechanism and demonstrated that the rate of stress increase due to water uptake exceeds the rate of stress relaxation in the cell wall (Ortega 2023). Furthermore, he showed that decreasing the rate of cell wall relaxation, proportional to the wall’s irreversible extensibility, increases turgor pressure (Ortega 2023).

In a series of studies, turgor pressure in the stage IVb sporangiophore of *P. blakesleeanus* was increased using a pressure probe device, and change in elongation growth rates was recorded (Ortega et al. 2003, 1989, 1991, 1995). A small step increase in turgor pressure (ΔP<0.02 MPa) produces an increase in the growth rate. However, larger increases in turgor pressure produce a decrease in growth (both elongation and rotation), ultimately yielding growth rates lower than the growth rates prior to pressurization, the pressure response. Interestingly, the $R_{tip}$ (ratio of rotation rate and elongation rate measured at the top of the growth zone, or tip) increased during the pressure response. The measured elongation and rotation rates before and after a step-up in turgor pressure of 0.066MPa are shown in Table 2. The transient increase in elongation rate, followed by a subsequent decline, is consistent with observations from pressure probe experiments conducted on other fungal species (Lew 2011).

**Table 2:** Tip elongation rate before and after a step-up in turgor pressure, *P*. Experimental results present the average values and the standard error calculated for 16 experiments (Ortega et al. 2003)

*P* (MPa)	$v_{tip}$ (experiments) μm/min	$\dot{\alpha }_{tip}$ (experiments) $(^{\circ }/min)$	$v_{tip}$ (model) μm/min	$\dot{\alpha }_{tip}$ (model) $(^{\circ }/min)$	$k_d$ (1/*min*)	$k_a$ (1/*min*)
0.349	45.9 ± 2.6	13.8 ± 0.7	48.7	13.2	0.21	0.14
0.416	19.0 ± 1.5	6.6 ± 0.5	20.7	8.3	0.04	0.01

The reduction in the tip rotation rate $\dot{\alpha }_{tip}$ suggests that changes in the deposition rate alone cannot account for the observed behavior; instead, the creep deformation of the sporangiophore under turgor pressure also contributes to this effect. Assuming that the non-dimensional tether stiffness $E^\star$, which depends on the material properties, remains unchanged, the increase in the shear to elongation rate ratio $R_{tip}$ indicates that the equilibrium constant $K^\star$ must decrease (Fig. 5C). This implies that the bond detachment to attachment rate ratio $k_d/k_a$ is decreasing. Given that the decrease in the growth rates, both elongation and rotation rates, is attributed to the decrease in the bond kinetics, we conclude that the decline in $k_a$ is more pronounced than that in $k_d$. Ortega demonstrated that an increase in irreversible wall extensibility leads to a reduction in turgor pressure (Ortega 2023), and that stress relaxation in the cell wall scales inversely with the irreversible wall extensibility. Showing the bidirectional relationship, our model predicts that the increased turgor pressure reduces the wall extensibility (or equivalently, the bond detachment rate). Furthermore, increased non-dimensional turgor pressure $\sigma ^\star$, and reduced non-dimensional equilibrium ratio $K^\star$ both indicate a smaller growth zone length, which is consistent with experimental findings. The model predicted values for the pressure response experiments are presented in Table 2.

**Fig. 6:**
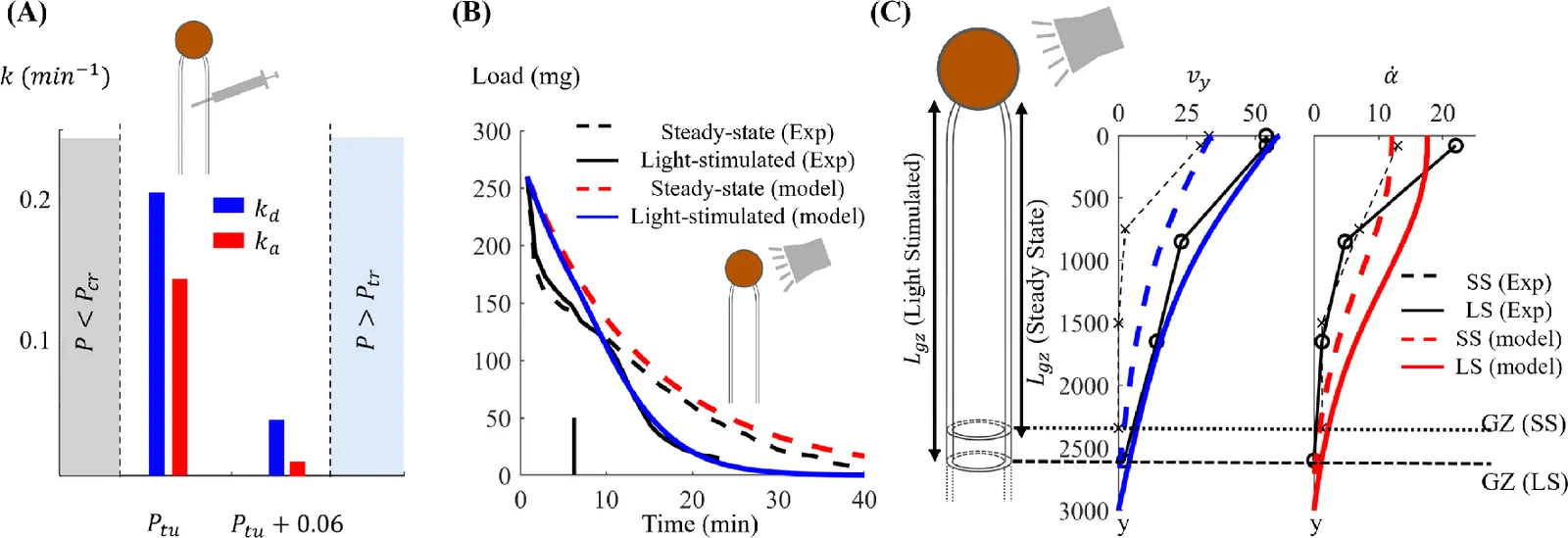
Stimulated growth. (A) Model prediction of kinetic growth in steady state and pressure response growth. Permanent growth occurs if the turgor pressure exceeds a critical value $P_{cr}$. Also, a catch-to-slip pressure $P_{cr}$ is predicted. Light-stimulated growth: (B) Load (mg) versus time (min) in a uniaxial tensile stress relaxation test for steady state (dashed) and light-stimulated (solid) sporangiophores. The black bar on the time axis marks the period of light exposure. (C) Elongation rate $v_y$ (μm/min) and rotation rate $\dot{\alpha }$ ($^\circ$/min) as functions of distance from the sporangium base *y* (μm), before and after light stimulation. black curves denote experimental data (Ortega 1976); colored curves indicate model predictions. Dashed curves correspond to the steady-state, and solid curves to light stimulation

The predicted decrease in the bond detachment rate $k_d$ after an increase in the force (due to turgor pressure), which implies longer lifetimes for the tether-fibril bonds, resembles that of a "catch bond" (Thomas et al. 2008), which has been observed in various length scales, from fire ant rafts(Wagner et al. 2024) to *E. Coli* Sokurenko (Evgeni et al. 2008). Therefore, to model the pressure response, the model must incorporate force-dependent kinetics in order to reconcile the datasets. One plausible mechanism underlying this force dependence is catch-bond–like behavior. Physical models utilizing the existence of energy wells, catch pathways, and slip pathways have been proposed for this phenomenon (Evans et al. 2004; Pereverzev Yuriy et al. 2005). Given the existence of a critical catch-to-slip transition force in these bonds, the bond dissociation rate $k_d$ will probably increase if the turgor pressure is increased further. However, further experiments are needed to evaluate the bond dynamics and the catch-bond–like behavior of the tether–fibril network. The catch-bond-like behavior is shown in Fig. 6A, and the pressure in the possible slip regime is shown with a blue background. Experimental support for the Lockhart growth equation suggests that expansive growth is initiated only when turgor pressure surpasses a yield threshold, a behavior analogous to that of a Bingham fluid. Although our current model does not explicitly incorporate a yield stress, all results presented are based on values of turgor pressures exceeding the experimentally established yield value. The critical pressure is reported to be approximately 0.26 MPa for stage IVb (Ortega et al. 1991) and is shown with a gray background in Fig. 6A. The incorporation of a critical pressure threshold remains an avenue for future investigation.

### Light response

The sporangiophore of *P. blakesleeanus* shows sensory responses to light stimulation. These responses include phototropism (growing toward or away from the light source), increased growth rates, and changes in the mechanical characteristics of the cell wall (Russo and Galland 2005; Eibel Grolig et al. 2000; Corrochano and Galland 2019; Ortega et al. 2021; Mohan 2019). We study how our model predicts the data on the light-stimulated experiments and relate the light-stimulation mechanism to the underlying physical kinetic quantity.

*Light-stimulated stress relaxation* Ortega conducted a tensile uniaxial stress-relaxation test on a light-stimulated sporangiophore (Ortega 1976). Similar to the steady-state experiment, the sporangiophore was stretched quickly to a force of 260 mg and held at that deformation. The sporangiophore was stimulated by a pulse-up light exposure for 45 seconds, 5 minutes after being fully stretched. The onset of light stimulation is depicted by a black box in Fig. 6B, where the force(mg) vs. time (min) is plotted for the steady state (dashed) and light-stimulated (solid) sporangiophore. The stress decay in the light-stimulated sporangiophore becomes more rapid, about 3-4 minutes after exposure to light stimulation. Since our model predicts that the relaxation rate only depends on the bond dissociation rate $k_d$, we conclude that light stimulation decreases the bond lifetimes. Furthermore, since the change in relaxation rate is delayed compared to the beginning of the light stimulation, we consider the following time dependency for the dissociation rate, $k_d$:

3.2 $$\begin{aligned} \dot{k}_d = \frac{-1}{\tau _l} \left( k_d^f - k_d^0 \right) \end{aligned}$$

where $\tau _l$ is a light-related time-scale and $k_d^f$ represents the final value of $k_d$. As depicted in Fig. 6B, the model captures the faster decay in the stress value, which is initiated about 4 minutes after the light exposure. The predicted initial and final dissociation rates are $k_d^0 = 0.08~\text {min}^{-1}$ and $k_d^f = 0.24~\text {min}^{-1}$, respectively. The characteristic timescale of the increase in the bond detachment rate is $\tau _l = 10~\text {min}$, indicating a delay on the order of several minutes in the change of bond kinetics following light stimulation.

*Light-stimulated growth* Helical growth of the sporangiophore of *P. blakesleeanus* can be altered during accelerated growth when exposed to light stimulation. Ortega (1976) measured the effects of increased growth rate due to light stimulation on the elongation and rotation rates along the stage IVb sporangiophore. In these experiments, the sporangiophore was light-adapted to a light source with an intensity of $1.4 \; mw/cm^2$ for 30 minutes. After measuring the elongation and rotation rates at various points, the sporangiophore was exposed to pulse-up light stimulation of 2 $mW/cm^2$ for one minute. Subsequently, the elongation and rotation rates at the same points were measured again. Fig. 6C illustrates the measured values of the elongation rates and rotation rates along the sporangiophore from these experiments. In addition to an increased length of the growth zone, light stimulation increased both tip elongation and rotation rates. Interestingly, the trend in rate decay changed in the light-stimulated sporangiophore. Unlike steady-state growth, the lower region of the growth zone in the light-stimulated sporangiophore exhibited elongation without significant rotation.

Previous studies have demonstrated that turgor pressure remains unchanged during light-stimulated growth (Ortega et al. 1988), allowing us to use the same value of $\sigma ^\star$ as in steady-state growth. Assuming that the intrinsic material properties of fibrils and tethers also remain constant (i.e., the same $E^\star$), we attribute the effects of light-stimulation to changes in the equilibrium constant $K^\star$ and the non-dimensional deposition rate $L^\star$. The observed reduction in the tip rotation-to-elongation ratio *R* under light stimulation implies an increase in the shear-to-elongation rate ratio $\dot{\gamma }/\dot{\varepsilon }$, which, according to Fig. 5B, corresponds to an elevated non-dimensional equilibrium constant $K^\star$. This suggests that the increase in the bond association rate $k_a$ surpasses the increase in the dissociation rate $k_d$. The latter is supported by the observed faster relaxation behavior discussed earlier. Therefore, during light-stimulated growth, the bonds not only have shorter lifetimes due to higher $k_d$, but also reattach more rapidly to the cell wall network. Furthermore, the longer growth zone observed experimentally under light stimulation implies an increased deposition rate *v*, as indicated in Fig. 5D. The corresponding model predictions for steady state (before) and light-stimulated (after) growth rates are presented in Fig. 6C. These steady-state predictions correspond to $k_d = 0.08 \text {min}^{-1}$ and $K^\star = 0.3$. The light-stimulated predictions correspond to $k_d = 0.16 \text {min}^{-1}$ and $K^\star = 0.4$. The influence of light stimulation on bond dynamics exhibits a more intricate behavior, as it varies with light intensity. The predicted variations in the kinetic rates during stimulated growth exhibit the same trend when force-dependent kinetic rates are used. Interestingly, following the transient increase in growth rate, the tip speed of the light-stimulated sporangiophore declines to values lower than its initial steady state, before recovering to steady-state levels(Ortega 1976).

## Discussion

In this work, we developed a dynamic model of the cell wall in the sporangiophore of *Phycomyces blakesleeanus* to reproduce experimental observations of active growth and passive mechanical tests. Inspired by the cell wall architecture, in which long chitin fibrils are crosslinked by chitin and chitosan tethers, cell wall anisotropy was represented through considering two stiffness values. $E_f$ represented the stiffness along the fibrils and is independent of the crosslinking density of tethers, and the transverse stiffness $FE_t$ was assumed to scale linearly with the fraction of attached tethers *F*. Using the transient network theory, we derived the governing equations, which are equivalent to those of a 2-dimensional Maxwell fluid with relaxation time $1/k_d$. We further introduced four nondimensional parameters: non-dimensional turgor pressure $\sigma ^\star$, non-dimensional tether stiffness $E^\star$, non-dimensional deposition rate $L^\star$, and non-dimensional equilibrium constant $K^\star$, and analyzed their effects on experimentally measurable quantities such as tip elongation and rotation rates, and length of the growth zone. We analytically demonstrated that the ratio of rotation to elongation rate at each material point depends on the non-dimensional transverse stiffness (ratio of transverse to longitudinal stiffness) $FE^\star$. Networks with greater fibril stiffness exhibit left-handed helical growth under constant turgor pressure, consistent with stage IVb, whereas networks stiffer in the transverse direction undergo right-handed growth. This framework explains the observed rotational inversions: right- to left-handed from stage IVa to IVb, and left- to right-handed from stage IVb to IVc, as arising from shifts in relative wall stiffness. In Goriely and Tabor (2011), it is assumed that new fibrils are secreted in the direction of existing ones, which differs from experimental evidence of implying the deposition of new fibrils in the transverse direction. Nonetheless, the transition from precompression to tension described in Goriely and Tabor (2011) can be regarded as a special case of our mechanism, since compressed fibrils would exhibit lower stiffness in their direction than stretched fibrils. According to our theory, fibrils undergo stretching and increased spacing during both right- and left-handed helical growth. However, the magnitude and relative pattern of these changes differ between the two scenarios.

Using the above analyses, we identified a feasible parameter set that fits the experimental data. We assumed constant bond kinetics ($k_a$, $k_d$) across space and time, employing stress-relaxation experiments to constrain the admissible range of $k_d$. The rotation-to-elongation ratio along the sporangiophore and the length of the growth zone were then used to estimate the transverse stiffness and deposition rate. As noted, we neglected the force dependence of the detachment rate $k_d$. Consequently, model predictions for the loading–unloading experiments (Norman et al. 1973) did not exactly reproduce the data, though they captured the experimental trends. Incorporating strain-hardening into the model is expected to improve the agreement. Through non-dimensional analyses, the model provides insight into the mechanisms driving altered growth behaviors, including those seen in pressure-probe and light-stimulation experiments. The accelerated relaxation observed in light-stimulated sporangiophores can be explained by an increase in the bond dissociation rate $k_d$. In contrast, the higher growth rate and extended growth zone length are linked to an increased equilibrium constant $K^\star$ and reduced non-dimensional deposition rate $L^\star$. Changes in turgor pressure also influence growth: a slight increase enhances the growth rate, whereas a drastic increase suppresses both elongation and rotation rates. By attributing these effects to bond dynamics, we predicted a catch-bond-like behavior for the bonds in the cell wall.

Using this model, we can rationalize how molecular mechanisms give rise to emergent growth behaviors in response to environmental and biochemical signals. Auxin (IAA), for instance, has long been recognized as a regulator of plant growth, and its effects on fungi, including *P. blakesleeanus*, have also been investigated (Živanović Branka et al. 2018; Ortega Joseph et al. 2015). Within our framework, increases or decreases in growth rate can be attributed to changes in bond kinetics within the polymer network. When interpreted alongside experimental results, such as stress-relaxation tests, the model allows for a more detailed understanding of how association and dissociation rates are modulated. Because of the shared fibrillar wall architecture, this framework is readily extendable to other fungal species and even plants. Such a generalization, together with the inclusion of additional mechanical processes (i.e., sporangiophore bending), remains a subject for future investigation.

## Supplementary Information

Below is the link to the electronic supplementary material.Supplementary file 1 (pdf 1184 KB).

## Data Availability

No datasets were generated or analysed during the current study.
