# Supplementary material for: Cell wall-driven mechanisms underlying emergent growth in phycomyces
Source: Biomech Model Mechanobiol. 2026 Jul 10;25(4):80. doi: 10.1007/s10237-026-02085-3 (PMC13354682; doi:10.1007/s10237-026-02085-3)
Supplement: Supplementary file 1 — (pdf 1184 KB). [file 10237_2026_2085_MOESM1_ESM.pdf]

Electronic Supplementary Information (ESI)

Cell Wall-Driven Mechanisms Underlying Emergent Growth in Phycomyces

Behnam Rezaei[a], Joseph. K. E. Ortega[b], and Frnack J. Vernerey[a,1]

[a]Department of Mechanical Engineering, The University of Colorado Boulder, Boulder, USA

[b]Department of Mechanical Engineering, University of Colorado Denver, Denver, CO 80217-3364, USA

[1]correspondence to: franck.vernerey@colorado.edu

1 Derivation of the model by the transient network theory

Assume a network of tethers with a population $N = cL_f$ between two parallel fibers, where L_f is the length of a fibril and c the number density of available tethers (per unit length). The end-to-end vector of each tether can be represented as $\boldsymbol{r} = [r, \Delta]^T$, where Δ is the distance between the two fibrils. We assume that the distribution of end-to-end vector $\phi(\boldsymbol{r}, t)$ of the fiber population can be decomposed as $\phi(\boldsymbol{r}, t) = F(t)p(\boldsymbol{r}, t)$ where $p(\boldsymbol{r}, t)$ is the probability of finding a particular fiber at the end-to-end configuration of $\boldsymbol{r}$ and f is the ratio of attached tethers to the total number of tethers $F = c/c_t$. With this definition, $F(t) = \int_\Omega \phi(\boldsymbol{r}, t) d\Omega$ where the integral is taken over all chain configurations. Assuming that total chains c_t remain constant, i.e., no damage is considered during deformation, we write the time evolution equation for ϕ through the Fokker-Planck equation [1] as:

$$\frac{D\phi}{Dt} = -\nabla \cdot (\phi \dot{\boldsymbol{r}}) + \xi_a - \xi_d \tag{1.1}$$

where ξ_a and ξ_d represent the source and sink terms due to bond attachments and detachments, respectively. The source and sink terms can be expressed in terms of kinetic rates k_a and k_d through:

$$\xi_a(\boldsymbol{x}, \boldsymbol{r}, t) \ = \ k_a(1 - F(\boldsymbol{x}, \boldsymbol{t}))p_a(\boldsymbol{x}, \boldsymbol{r}, t) \quad \text{and} \quad \xi_d(\boldsymbol{x}, \boldsymbol{r}, t) \ = \ k_d(\boldsymbol{x}, r)F(\boldsymbol{x}, t)p(\boldsymbol{x}, \boldsymbol{r}, t) \tag{1.2}$$

where we assumed that the ratio $(1 - F)$ of unattached tethers connect to the network with an attachment probability of p_a and attachment rate k_a. Furthermore, it is assumed that the attached tethers with a ratio of F detach from the network in the current state of the network and with a detachment rate of k_d. By integrating Eq. 1.2 over the chain configuration, and using an affine assumption, where the tethers follow the macroscopic deformation, the evolution equation of the ratio F of the attached tethers is calculated as:

$$\dot{F} = k_a(1 - F) - k_d F. \tag{1.3}$$

The above equation is derived for constant values of k_a and k_d, which represent the average attachment and detachment rates over the population of tethers. For more details on the above derivation and the integral calculations, see [1, 2]. Additionally, we can calculate the evolution equation of the covariance matrix, which is defined as:

$$\boldsymbol{M}(\boldsymbol{x}, \boldsymbol{r}, t) = \int_\Omega p(\boldsymbol{x}, \boldsymbol{r}, t)\,(\boldsymbol{r} - \langle \boldsymbol{r} \rangle) \otimes (\boldsymbol{r} - \langle \boldsymbol{r} \rangle)\; d\Omega = \int_\Omega p(\boldsymbol{x}, \boldsymbol{r}, t)\, \boldsymbol{r} \otimes \boldsymbol{r}\; d\Omega \tag{1.4}$$

where we assumed that $\langle r \rangle = 0$, which is valid for large population of tethers. By multiplying Eq. 1.2 by $\boldsymbol{r} \otimes \boldsymbol{r}$, and then integrating it over the chain configuration, the evolution equation of the covariance tensor

$\dot{M}$ is calculated as:

$$\dot{\boldsymbol{M}} = \boldsymbol{\ell M} + \boldsymbol{M \ell}^T - k_a \left(1 - \frac{1}{F}\right) \boldsymbol{M}_a - \int_\Omega k_d \, p \, \boldsymbol{r} \otimes \boldsymbol{r} \, d\Omega - \frac{\dot{F}}{F}\boldsymbol{M} \tag{1.5}$$

where $\boldsymbol{\ell}$ is the velocity gradient tensor, and $\boldsymbol{M}_a = \int_\Omega p_a \, \boldsymbol{r} \otimes \boldsymbol{r} \, d\Omega$ is attached configuration of the network. For more details about the above derivation, see [1, 2]. In the case of attached tethers between two fibers under shear loading, we are interested in the shear element $\boldsymbol{M}_{12}$ of the covariance matrix. Thus, $\dot{\langle r \rangle} = \dot{\boldsymbol{M}}_{12}/\Delta$ is then calculated as:

$$\dot{\langle r \rangle} \; = \; \dot{\gamma} + k_a \left(1 - \frac{1}{F}\right) (\langle r \rangle - \langle r_a \rangle) \tag{1.6}$$

We assume that the tethers reattach to the network in a stress-free configuration, i.e., $\langle r_a \rangle = 0$. This assumption satisfies the Clausius–Duhem inequality (second law of thermodynamics) [1]. Further using the simplifying assumption that tethers are linearly elastic, with stiffness K, the stress over the full population is given be $\tau = c_a K r = F c_t K r$, the above equation can be rewritten as:

$$\dot{\tau} + k_d \tau \; = \; F c_t K \dot{\gamma} \tag{1.7}$$

2 Analytical expressions for shear and longitudinal Deformation Rates

In modeling growth, the deformation is the response of the model to a creep experiment where a constant stress is applied ($\dot{\boldsymbol{\sigma}} = 0$). In the absence of an axial moment, we assume that the shear stress component associated with the twist in helical growth, $\sigma_{12} = 0$. Therefore, the only nonzero elements of the stress tensor are σ_{11} and σ_{22}, which correspond to the Hoop stress, and longitudinal stress, respectively. By applying the inextensibility condition in the radial direction ($\varepsilon_{11} = 0$), the relation between these two stresses can be calculated. To better understand the effects of each parameter on the growth model, we calculate the deformation rates for an element whose fibrils are oriented with an angle θ with respect to the horizontal axis. Therefore, small values of θ correspond to the upper regions of the growth zone, while larger values (approaching 90 degrees) correspond to the lower regions of the growth zone. The vector of transverse isotropy can be represented as $\boldsymbol{a} = [\cos\theta \;\; \sin\theta]^T$. Given the introduced nondimensional parameters, the expression for the longitudinal $\dot{\varepsilon}$ and shear $\dot{\gamma}$ deformation rates degenerate to:

$$\dot{\varepsilon} \; = \; \frac{1 + 3FE^\star + (FE^\star - 1)\cos(4\theta)}{4FE^\star \left(FE^\star + (FE^\star - 1)(\sin^4(\theta) - 2\sin^2(\theta))\right)} k_d \sigma^\star \tag{2.1}$$

$$\dot{\gamma} \; = \; \frac{(FE^\star - 1)\,(2\sin(2\theta) - \sin(4\theta))}{4FE^\star\,(1 + (FE^\star - 1)\cos^4(\theta))} k_d \sigma^\star \tag{2.2}$$

and the ratio between these two deformation rates degenerates to:

$$\frac{\dot{\gamma}}{\dot{\epsilon}} = \frac{(FE^\star - 1)\,(\sin(2\theta) - \cos(2\theta)\sin(2\theta))}{\sin^2(2\theta) + FE^\star(1 + \cos^2(2\theta))} \tag{2.3}$$

From Equation 2.2, it is clear that the direction of the twist, which can be related to the sign of $\dot{\gamma}$, depends on the value of $(FE^\star - 1)$. If the network exhibits greater stiffness in the transverse direction ($FE^\star > 1$), resulting in a positive value of $\dot{\gamma}$, the helical growth is right-handed (counterclockwise). Conversely, when the network is stiffer along the fibril direction, the helical growth becomes clockwise (left-handed).

Interestingly, Equation 2.2 predicts a non-monotonic relationship between the rotation and the inclination angle as the latter increases. In the general case, determining the extremum point (the angle at which the

magnitude of the shear rate is maximized) requires knowledge of the dependencies of k_d and F on the inclination angle θ. However, in the case of constant parameters, the extremum point is found by solving $\frac{\partial \dot{\gamma}}{\partial \theta} = 0$. The solution to this equation depends on the value of $FE^\star$. Furthermore, simplifications lead to:

$$\sin^2(\theta) = \frac{6FE^\star - 2 \pm \sqrt{3FE^\star + 1}}{3FE^\star - 3} \tag{2.4}$$

Figure S1. illustrates the changes in the shear rates, deformation rates, and their ratio as vs. the values of $FE^\star$ for different values of θ, and the same rates vs. the inclination angle θ for different values of $FE^\star$.

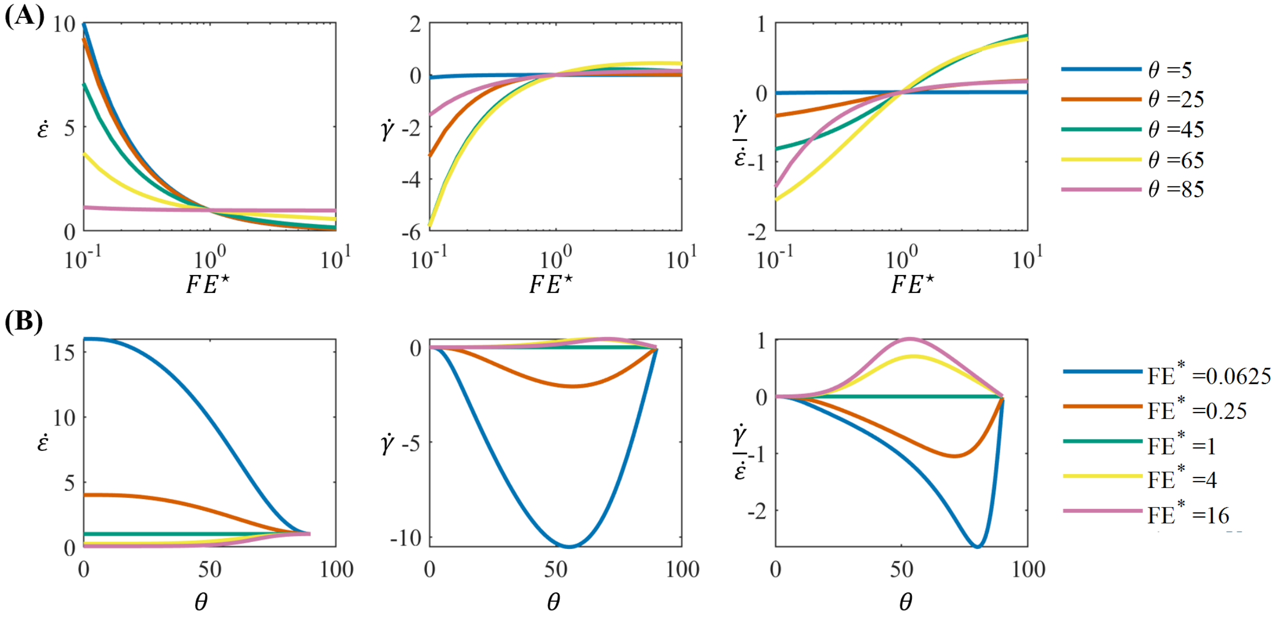

Fig. S1. (A) Elongation rate $\dot{\varepsilon}$, rotation rate $\dot{\gamma}$ and their ratio vs. the normalized transverse stiffness for inclination angles $\theta = 5°,\ 25°,\ 45°,\ 65°,$ and $85°$. (B) Elongation rate $\dot{\varepsilon}$, rotation rate $\dot{\gamma}$, and their ratio vs. the inclination angle for normalized transverse stiffness $FE^\star = 1/16, 1/4,\ 1,\ 4,$ and 16.

3 Parameter sensitivity analysis

The growth model predictions, elongation rate v_y and rotation rate $\dot{\alpha}$, are shown for various values of the equilibrium constant $K^\star$, non-dimensional tether stiffness $E^\star$, and nondimensional turgor pressure $\sigma^\star$ (Figure S2). In contrast to these nondimensional parameters, the nondimensional deposition rate $L^\star$, which represents the deposition rate, only scales the growth rate distributions, and doesn't affect the creep simulation output. Since our fitting process is based on manual tuning, $L^\star$ can be adjusted so that qualitatively similar fits are produced for various values of the nondimensional turgor pressure $\sigma^\star$, the nondimensional tether stiffness $E^\star$, and the equilibrium constant $K^\star$.

4 Definition of the growth zone

As discussed in the main text, the criterion for determining the length of the growth zone L_{gz} is defined as the material point at which both the elongation and rotation rates drop to a fixed fraction of their respective maximum values (one percent in our simulations). This criterion was chosen based on the observation that material points may exhibit a nonzero elongation rate with negligible rotation, or vice versa. Alternatively,

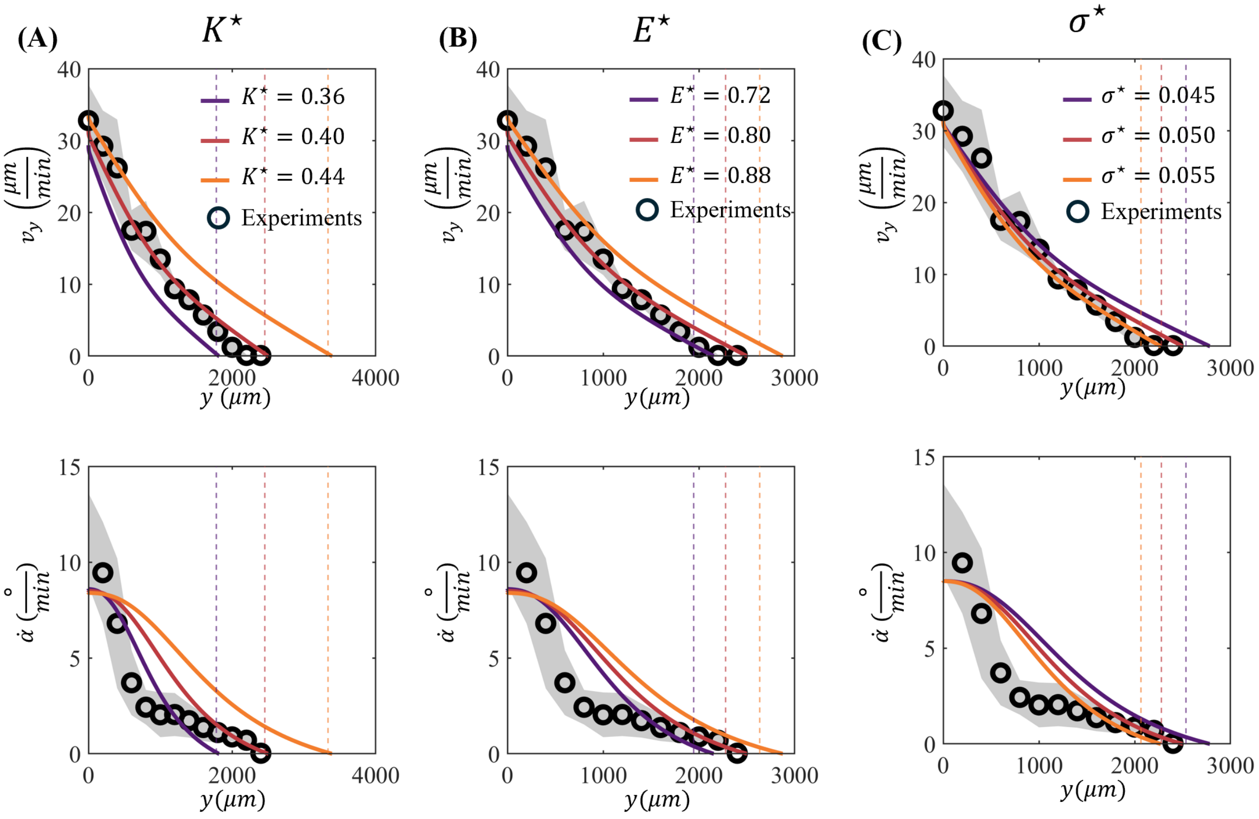

Fig. S2. Parameter sensitivity analysis: Nondimensional elongation rate v_y/v, nondimensional rotation rate $R_s\dot{\alpha}/v$, and the rotation rate to elongation rate ratio $\dot{\alpha}/v_y$ as a function of nondimensional distance from sporangium y/L for (A) $K^\star = 0.3$, 0.4, and 0.5. (B) $E^\star = 0.4$, 0.6, and 0.8. (C) $\sigma^\star = 0.03$, 0.05, and 0.07. The end of the growth zone is determined using the dashed lines.

the length of the growth zone can be defined using only one of these criteria, or as the last material point at which the fiber orientation is nearly vertical ($\theta \approx 90^\circ$). While the choice of criterion affects the absolute value of the growth-zone length, it does not alter the trends in how the growth zone varies with the introduced parameters. Figure S4 illustrates the elongation and rotation rates for a representative simulation, in which the last material point with nearly vertical fiber orientation is located 2500 μm from the sporangium. The boundaries of the growth zone, defined based on the maximum length, where both ending criteria are met (dashed line), and the minimum length, where only one ending criterion is met (dotted line), are indicated in the figure. The predicted growth zone lengths obtained using these different criteria are of the same order of magnitude.

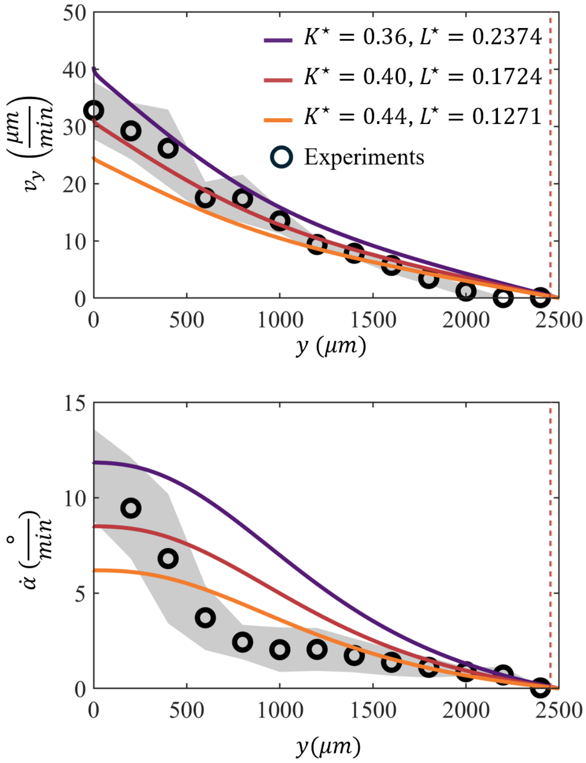

Fig. S3. Qualitatively close fits for the experimental data on growth rates using three sets of values for the equilibrium constant $K^\star$ and nondimensional deposition rate $L^\star$.

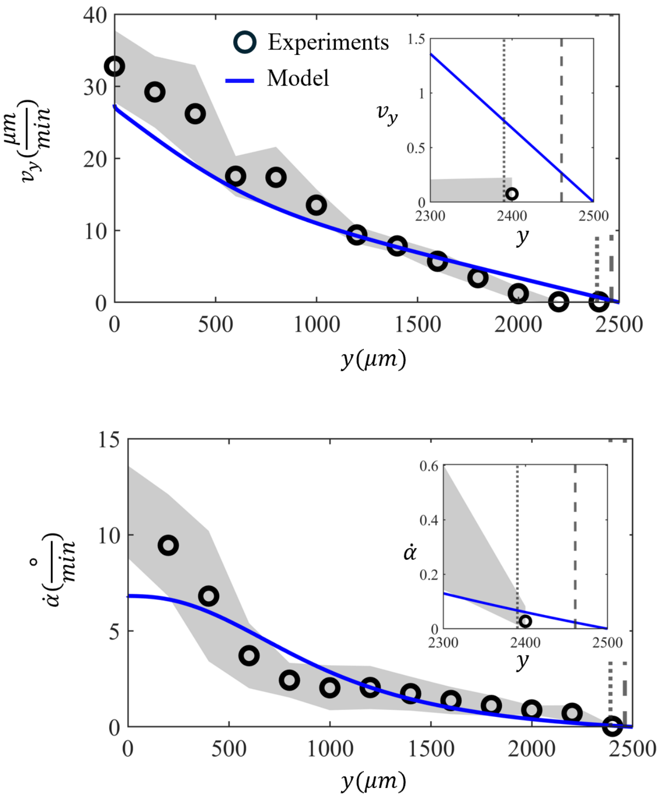

Fig. S4. Definition of the growth zone based on maximum length satisfying both fraction conditions (dashed line) and minimum length satisfying only one of the conditions (dotted line) vs. the end of the growth zone based on the final angle θ_{final}. (Inset): magnification of the graphs between 2300 $\mu m \leq y \leq 2500$ μm

References

[1] Franck J Vernerey, Rong Long, and Roberto Brighenti. A statistically-based continuum theory for polymers with transient networks. *Journal of the Mechanics and Physics of Solids*, 107:1–20, 2017.

[2] Franck J Vernerey, Behnam Rezaei, and Samuel C Lamont. A kinetic theory for the mechanics and remodeling of transient anisotropic networks. *Journal of the Mechanics and Physics of Solids*, 190:105713, 2024.
